# Characterizing the Large‐Scale Structure of Multimodal Semantic Networks

**DOI:** 10.1111/cogs.70131

**Published:** 2025-10-23

**Authors:** Raja Marjieh, Pol van Rijn, Ilia Sucholutsky, Harin Lee, Nori Jacoby, Thomas L. Griffiths

**Affiliations:** ^1^ Department of Psychology Princeton University; ^2^ Max Planck Institute for Empirical Aesthetics; ^3^ Center for Data Science New York University; ^4^ Max Planck Institute for Human Cognitive and Brain Sciences; ^5^ Department of Psychology Cornell University; ^6^ Department of Computer Science Princeton University

**Keywords:** Semantic networks, Perception, Naturalistic stimuli, Language

## Abstract

Humans organize semantic knowledge into complex networks that encode relations between concepts. The structure of those networks has broad implications for human cognitive processes, and for theories of semantic development. Evidence from large lexical networks such as those derived from word associations suggest that semantic networks are characterized by high sparsity and clustering while maintaining short average paths between concepts, a phenomenon known as a “small‐world” network. It has also been argued that those networks are “scale‐free,” meaning that the number of connections (or degree) between concepts follows a power‐law distribution, whereby most concepts have few connections, while a few have many. However, the scale‐free property is still debated, and the extent to which the lexical evidence reflects the naturally occurring semantic regularities of the environment has not been investigated systematically. To address this, we collected and analyzed semantic descriptors, human evaluations, and similarity judgments from four large datasets of naturalistic stimuli across three modalities (visual, auditory, and audio‐visual) comprising 7916 stimuli and 610,841 human responses. By connecting concepts that co‐occur as descriptors of the same stimuli, we construct “multimodal” semantic networks. We show that these networks exhibit a clear small‐world structure with a degree distribution that is best captured by a truncated power law (i.e., the most‐connected concepts are less common than predicted by a perfect power law). We further show that these networks are predictive of human sensory judgments on these domains, as well as reaction times in an independent lexical decision task. Finally, we show that multimodal networks also share overlapping themes with previously analyzed lexical networks, which upon a more rigorous reanalysis are revealed to be truncated too. Our findings shed new light on the origins of the structure of semantic networks by tying it to the semantic regularities of the environment.

Humans rely on rich representations of conceptual knowledge to effectively process the world around them and to facilitate inference, decision‐making, and memory retrieval (Abbott, Austerweil, & Griffiths, 2015; Collins & Quillian, 1969; Collins & Loftus, 1975; De Deyne & Storms, 2008; De Deyne, Navarro, Perfors, Brysbaert, & Storms, 2019; Griffiths, Steyvers, & Tenenbaum, 2007; Griffiths, Steyvers, & Firl, 2007; Jones, Gruenenfelder, & Recchia, 2018; Lynn & Bassett, 2020). These representations organize and encode the relationships between concepts, which are generally referred to as *semantic networks*. Understanding the structure of semantic networks, their development, and the computations that they support has been central to the field of cognitive science for decades (Borge‐Holthoefer & Arenas, 2010; Collins and Quillian, 1969; Collins & Loftus, 1975; Hills, Maouene, Maouene, Sheya, & Smith, 2009; Steyvers & Tenenbaum, 2005; Siew & Vitevitch, 2020a). Research on semantic networks has been further enriched by developments in other domains such as dimensionality reduction (Landauer & Dumais, 1997), network science (Steyvers and Tenenbaum, 2005; Borge‐Holthoefer and Arenas, 2010), search algorithms (Griffiths et al., 2007), holographic representations (Jones & Mewhort, 2007), topic models (Griffiths et al., 2007), and deep learning (Manning, Clark, Hewitt, Khandelwal, & Levy, 2020; Peterson, Chen, & Griffiths, 2020), each providing hypotheses and data structures against which human behavior could be evaluated.

Early seminal work on semantic networks focused on the design of network structures that could support operations such as effective memory search and fact verification. For example, Collins and Quillian (1969) proposed a hierarchical semantic network (see Fig. 1 for an example schematic) to capture animal taxonomies and their properties. The top of the hierarchy (center node in Fig. 1) corresponds in that case to the category *Animal* along with its core properties (e.g., “has skin,” “can eat”), and then each branch (or edge) to other concepts (or nodes) corresponds to an inclusion relation (e.g., *Animal* → *Bird*, *Animal* → *Fish*) which, consequently, get refined further down the hierarchy (e.g., *Bird* → *Canary*, *Bird* → *Ostrich*). To ensure that the representations were economical, properties stated higher in the hierarchy (e.g., “has wings” for *Bird*) are not repeated for the lower nodes (e.g., *Canary*). Given this structure, Collins and Quillian (1969) proposed a search algorithm that leverages its properties to verify statements such as “Canary has wings,” which they then showed that it can predict human reaction time in such tasks.

**Fig. 1:**
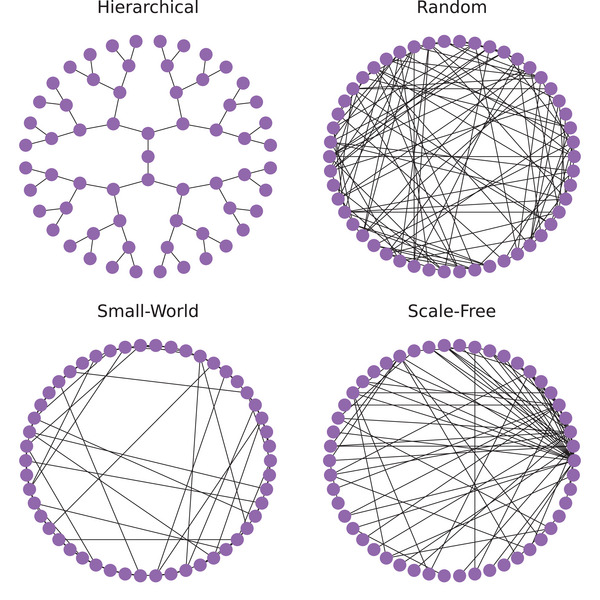
Different network organizations: hierarchical, random, small‐world, and scale‐free.

A few decades later, Steyvers and Tenenbaum (2005) offered a new angle on semantic networks by examining the structural properties of lexical networks such as those derived from word association tasks (i.e., two words are connected if one is elicited as a free association of the other, e.g., *sky* → *blue*), and attempting to explain those properties as the product of cognitive developmental processes. The authors found that lexical semantic networks are characterized by sparse connectivity (i.e., the chance of two generic concepts being connected is low on average), high clustering (i.e., if two concepts are connected to a third concept, then they are likely to be connected themselves by an edge), and short average path length (i.e., it is possible to reach from one concept to another by following a small number of edges on average). Such networks are known as *small‐world* networks (Watts & Strogatz, 1998), and they have drawn considerable attention over the years (Dorogovtsev & Mendes, 2002) due to their ubiquity, and the way they interpolate between order (high clustering) and randomness (short average paths) by relying on “shortcut” edges (Fig. 1). Steyvers and Tenenbaum (2005) further noted that the degree distribution (i.e., the distribution of the number of connections per word) of these networks admits a power‐law form (i.e., most words have a few connections, and a small fraction of “hubs” have many), a phenomenon known as a *scale‐free* network (Barabási, 2009; Fig. 1).

From an information processing perspective, these observations are significant. First, small‐world organization ensures search efficiency by keeping related concepts clustered, while also allowing for fast traversal of the network via shortcuts. Second, scale‐free network structure constrains the kinds of developmental processes underlying semantic organization. Indeed, to explain these properties, Steyvers and Tenenbaum (2005) proposed a model of semantic growth in which new concepts are added to a semantic network through a “conceptual differentiation” mechanism, whereby a new concept inherits a subset of the connections of an existing concept, with that existing concept being chosen with probability proportional to its number of connections, a process known as preferential attachment (Barabási & Albert, 1999; Dorogovtsev & Mendes, 2002). Finally, the small‐world and scale‐free properties are inconsistent with the hierarchical organization proposed by Collins and Quillian (1969), as such hierarchical networks have no clustering and no hubs (since the neighbors of a node are never connected themselves, and each node has a small number of connections; see Fig. 1).

While compelling, subsequent research revealed a more nuanced picture. First, conceptual differentiation is only one possible mechanism for producing scale‐free and small‐world networks (Borge‐Holthoefer and Arenas, 2010; Hills et al., 2009; Jones et al., 2018). For example, Hills et al. (2009) analyzed longitudinal data of early noun learning and argued that it was better captured by a preferential *acquisition* mechanism, whereby the order of nouns learned depends on the number of their connections (degree) in an *external* learning environment (e.g., the semantic network of adult utterances). This contrasts the model of Steyvers and Tenenbaum (2005), where the order of the learned nouns depends on the node degrees of the child's *internal* developing semantic network. Second, some analyses of lexical networks seem to suggest deviations from scale‐free properties (Siew and Vitevitch, 2020a; Siew & Vitevitch, 2020b; Utsumi, 2015). For example, by analyzing phonological networks (i.e., words are connected if they share similar sounds), Siew and Vitevitch (2020a) provided evidence for a non‐scale‐free structure that is best captured by a mixture of mechanisms. Finally, later advances in network science (Clauset, Shalizi, & Newman, 2009) made a strong argument that simple regression analyses for evaluating scale‐free properties (as used in Steyvers and Tenenbaum (2005)) may be inadequate due to statistical fluctuations, and more rigorous techniques have been proposed.

Perhaps most importantly, all aforementioned studies rely on the analysis of highly curated lexical datasets. The existing treatment in the literature does not consider an important organizing principle underlying semantic networks, namely, that they are adapted to the sensory environment and thus should reflect its regularities.

To highlight this point, consider the concept *sky* or the color *green*. These are highly recurrent features of natural scenes and thus are expected to co‐occur frequently with a variety of more specialized concepts like specific animals or vegetation. Likewise, scenes of everyday life often include humans performing a variety of activities and so one would expect concepts like *man* and *woman* to be highly prevalent in verbal descriptions of such scenes. The fact that humans are constantly processing the environment around them, and actively communicating useful information about it, raises the possibility that the extent to which concepts co‐occur in such environments, which can be used to construct *multimodal* ^1^ semantic networks (i.e., words are connected if they co‐occur as descriptors of the same stimulus), are internalized through a mechanism of statistical learning (e.g., by learning to extract the semantic gist of an observed stimulus; Griffiths et al. (2007)). Characterizing the structure of multimodal semantic networks is thus key to evaluating this complementary hypothesis concerning the sources of structure in human semantic networks. To the best of our knowledge, a systematic investigation into this problem has not been carried out.

To address this gap, we leveraged recent advances in online semantic mining techniques along with the growing availability of rich multimodal and naturalistic stimulus datasets. Specifically, we used a modern semantic mining procedure known as STEP‐Tag (Marjieh et al., 2023), whereby humans collaboratively and iteratively tag stimuli with semantic descriptors and evaluate the quality of tags proposed by others (Fig. 2A). The STEP‐Tag approach draws inspiration from the literature on *algorithms with people*, in which behavioral experiments are designed such that they incorporate human decisions in computer algorithms for characterizing representations (Harrison et al., 2020; Sanborn & Griffiths, 2007). In a STEP‐Tag process, a participant is presented with a stimulus (e.g., an image) and is asked to provide tags that describe it (e.g., “pizza” and “scoop”). The next participant then observes the stimulus along with the previous tags, evaluates their relevance, and contributes new tags. The process then repeats, and poor quality tags get filtered out (see Methods). This self‐regularizing feature of STEP‐Tag ensures that it converges on a set of high‐quality tags that capture the semantic content of each stimulus (Fig. 2B). By analyzing the co‐occurrence patterns of these lists of descriptors, we then constructed multimodal semantic networks that are associated with each stimulus domain (i.e., by connecting words that co‐occur as descriptors of the same stimulus; Fig. 2C). As a control, we complemented this approach with a more traditional one, whereby participants were asked to freely caption the stimuli they observed. As for the stimulus datasets considered, here we chose to cover a variety of domains that are of interest to different disciplines, including neuroscience, psychology, and machine learning. These datasets range from natural scenes (Chang et al., 2019) and modern artworks (Mohammad & Kiritchenko, 2018a) (visual), to emotional prosody recordings (Livingstone & Russo, 2018) (auditory), and video clips of everyday activities (Xie, Sun, Huang, Tu, & Murphy, 2018) (audio‐visual). Overall, our four behavioral datasets comprised 7916 stimuli and 610,841 human responses.

**Fig. 2:**
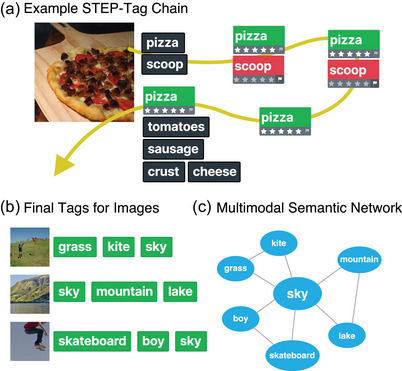
Behavioral paradigm and example stimuli. **(**A) Schematic of the STEP‐Tag semantic mining procedure. In this example, the first participant proposed the tags “pizza” and “scoop.” In the second iteration, “pizza” received a five‐out‐of‐five relevance rating by a second participant, whereas “scoop” got flagged. The same happened with the third participant, which led to the removal of “scoop” and so on. (B) Example natural image stimuli along with final tag descriptors. (C) The corresponding multimodal semantic network. *Note*. Sample images are reproduced from the BOLD5000 dataset of Chang et al. (2019).

Given the multimodal networks, we then subjected them to rigorous statistical analyses to evaluate whether they were small‐world and scale‐free. This was done by computing different network statistics, such as clustering measures and average shortest path lengths, as well as using log‐likelihood‐based methods for evaluating power‐law degree distributions and comparing them against competing models (Alstott, Bullmore, & Plenz, 2014; Clauset et al., 2009; see Methods). We also investigated the semantic content of these networks by analyzing their most connected words, in addition to using modern community detection algorithms to uncover thematic clusters (see Methods).

In addition to the structural analysis which forms the main contribution of the present work, we followed with a series of behavioral validation studies that are designed to evaluate whether the multimodal networks are compatible with other independent psychological data. First, we tested whether the derived descriptors could predict human pairwise similarity judgments over each of the domains (e.g., “how similar are the two images on a scale from 0‐1?”). Similarity judgments have a long history in cognitive science and are highly diagnostic of representations (Shepard, 1980; Tversky, 1977), and so it is natural to ask whether our elicited descriptors can predict such data. We produced numerical predictions from the descriptors by embedding them using a suitable text embedding model and then translating those embeddings into a similarity measure (see Methods). Second, we explicitly evaluated whether our derived networks overlapped with lexical networks by reanalyzing two well‐studied lexical networks that were considered by Steyvers and Tenenbaum (2005), namely, free associations (Nelson, McEvoy, & Schreiber, 2004) and WordNet.^2^ Finally, following Steyvers and Tenenbaum (2005), we also tested whether the degree of connectivity of words in the multimodal and lexical networks were predictive of the reaction time needed for a human to identify them correctly in a lexical decision task (Balota et al., 2007).

In the remainder of the paper, we present a systematic evaluation of the properties and behavioral relevance of multimodal semantic networks, which we derive directly from rich sensory data. The paper proceeds as follows: in the next section, we survey the technical details of all datasets, experiments, and analysis techniques described above. We then proceed to the Results, where we detail the properties of the derived multimodal networks, their semantic content and statistics, which, in turn, are followed by the behavioral evaluations. We then conclude with a Discussion.

## 1 Methods

### 1.1 Stimuli

In choosing the stimulus datasets, we aimed for the following: (i) naturalistic and complex domains; (ii) datasets that are relevant to multiple disciplines; (iii) covering different perceptual modalities; and (iv) covering both natural and abstract stimuli. Overall, we considered four datasets, which we describe below. Example stimuli are provided in the OSF repository (see Transparency and Openness).

**BOLD5000**. A collection of 4916 unique images of indoor/outdoor activities as well as natural scenes from the computer vision and neuroscience literature (Chang et al., 2019). This dataset is particularly relevant for the neuroscience community as it also contains slow‐event functional magnetic resonance imaging (fMRI) data from four subjects each observing 5254 images across 15 scanning sessions. The dataset was constructed such that it contains a diverse array of visual domains, including objects interacting in real‐world environments and scenes from daily life in order to capture a wide array of visual features, categories, and semantics (Chang et al., 2019). The images can be further grouped into: (i) 1000 hand‐curated indoor and outdoor scenes from 250 categories (Xiao, Hays, Ehinger, Oliva, & Torralba, 2010; the full list is provided in Supplementary Appendix A); (ii) 2000 images from the COCO dataset (Lin et al., 2014), depicting multiple objects (both inanimate and animate) and interactions between them (e.g., everyday human social interactions); and (iii) 1916 images from ImageNet of individual objects (Deng et al., 2009). In what follows, we will refer to this dataset as BOLD5K for brevity.

**Mini‐Kinetics**. A collection of 1000 short video clips of everyday activities from 200 activity classes taken from the Mini‐Kinetics‐200 dataset (Xie et al., 2018), which itself is a subset of the larger DeepMind Kinetics video dataset (Kay et al., 2017). The videos were sourced from YouTube and lasted about 10 s each. The activity classes encompass varied themes such as various sports activities, musical instrument playing, animal feeding, vehicle driving, eating, and so on (the full list is provided in Supplementary Appendix A). To maximize diversity, five random videos were sampled from each of the 200 activity classes. This set was originally constructed in Marjieh et al. (2023).

**WikiArt**. A collection of 1000 artworks from the WikiArt Emotions dataset (Mohammad & Kiritchenko, 2018b) comprising a variety of modern and abstract art paintings. Specifically, we randomly sampled 100 images from each of the following categories: Impressionism, Neo‐Expressionism, Post‐Impressionism, Cubism, Abstract Expressionism, Minimalism, Color Field Painting, Art Informel, Abstract Art, and Lyrical Abstraction. We chose these categories as we expected them to be more abstract.

**Emotional Prosody**. A collection of 1000 audio recordings from the RAVDESS corpus (Livingstone & Russo, 2018), which comprises a set of semantically neutral sentences spoken by 24 North American actors (gender‐balanced) to convey different target emotion expressions (calm, happy, sad, angry, fearful, surprise, and disgust) in addition to a neutral expression. The sentences were scripted and corresponded to “kids are talking by the door,” and “dogs are sitting by the door.” The subset of 1000 recordings was constructed in Marjieh et al. (2023) by randomly selecting three emotions per speaker per sentence, randomly omitting 104 emotional stimuli and including all 96 neutral recordings.

### 1.2 Participants

Participants were recruited online through Amazon Mechanical Turk (AMT). All participants provided informed consent prior to participation in accordance with approved Institutional Review Board protocols. To ensure data quality, participants had to reside in the United States, to be at least 18 years of age, and to have successfully completed at least 5000 tasks on AMT with an approval rate of 99%. We based these criteria on prior work to maintain high data quality on AMT (Hardy, Thompson, Krafft, & Griffiths, 2023). Participants additionally had to pass a standard English proficiency test in order to participate (LexTALE; Lemhöfer & Broersma (2012); details below). Overall, N=1918 participants took part in the STEP‐Tag paradigm, N=986 in the free caption control, and N=1224 in the similarity judgment paradigm (see details below). Demographic information is provided for all experiments in Supplementary Table L1.

**LexTALE Prescreener**. LexTALE is a common English proficiency test that was developed by Lemhöfer and Broersma (2012). In this test, participants were briefly presented with either real English words or nonwords (each word was presented for 1 s), and they had to decide whether the word was real or not. Participants were presented with 12 unique words (six real and six made up) and they had to give at least eight correct guesses to pass. The words used were: hasty, fray, kilp, plaintively, stoutly, moonlit, mensible, crumper, plaudate, alberation, scornful, unkempt. Overall, 804 participants failed to pass the test. Note that the reported numbers in the Participants section and in Supplementary Table L1 are the final numbers after excluding those who failed the prescreening task, as these participants were not allowed to proceed further and so did not participate in the main task.

### 1.3 Procedure

**STEP‐Tag**. The Sequential Transmission Evaluation Pipeline (STEP) for semantic tag mining (STEP‐Tag) was first introduced in Marjieh et al. (2023). In each trial of the paradigm, participants were presented with a stimulus (e.g., a video excerpt; Fig. 2A). If this was the first time the stimulus was presented, participants were asked to provide at least one tag (e.g., “type in words describing the activity in the video”; exact prompts for all domains and paradigms are provided in Supplementary Appendix B). If other participants already tagged the stimulus, the current participant was first asked to rate the relevance of the previous tags on a 5‐Likert scale or flag them if they seemed inappropriate (“you should rate the relevance of each tag by clicking the appropriate amount of stars (1 star not very relevant, 5 stars very relevant). If you think that the tag is a mistake or completely irrelevant, you should flag it by clicking the flag icon”). A tag that was flagged twice was removed from the list. Participants were then allowed to contribute additional tags if they wanted (e.g., “You can also add your own tag that is relevant to describe the activities in the video. Your tag will then be rated by other players who are playing the game simultaneously”). If participants flagged two or more tags by the same participant, the experiment ended early for that participant. Each STEP‐Tag process ran for a minimum of 10 iterations per stimulus and no more than 20 iterations. After 10 iterations, the process could terminate early (converge) if in the latest iteration there were at least two tags that were rated at least three times with a mean rating of three stars. In practice, we found that all processes associated with our stimuli converged within 10–16 iterations. The STEP‐Tag data for Mini‐Kinetics and Emotional Prosody were collected by Marjieh et al. (2023) and involved N=221 and N=217 participants, respectively. The STEP‐tag data for BOLD5K and WikiArt are newly collected and involved N=1223 and N=257 participants, respectively. Demographic information and additional descriptive statistics are provided in Supplementary Tables L1 and M1, respectively.

**Free Captions**. To ensure that any observed regularities in the semantic networks are not simply an artifact of the STEP‐Tag procedure, we additionally collected free captions over each of the stimulus sets. Specifically, in each trial, participants were presented with a random stimulus and were asked to freely describe it with words (e.g., “you will be presented with different videos of activities and your task will be to describe their content”). To prevent participants from providing very short or empty responses, we required that each description must contain at least five words, and a minimum of four unique words. The free caption data for Mini‐Kinetics and Emotional Prosody were collected by Marjieh et al. (2023) and involved N=196 and N=151 participants, respectively. The free caption data for BOLD5K and WikiArt are newly collected and involved N=410 and N=229 participants, respectively. Demographic information and additional descriptive statistics are provided in Supplementary Tables L1 and M2, respectively.

**Similarity Judgments**. To evaluate the extent to which the textual descriptors (both tags and captions) were predictive of the properties of the datasets, we compared them against similarity judgments over random subsets of 100 stimuli from each dataset. Similarity judgments are particularly interesting because they provide a quantitative way for evaluating how participants perceive and represent a certain stimulus domain without explicitly asking them to describe their perceptions in words. Instead, participants rate the perceived similarity between different pairs of stimuli on a numerical scale. By aggregating such pairwise judgments, one can construct a “similarity matrix” $s_{ij}$ that summarizes the perceived similarity between any pair of stimuli i and j. This idea has a long history in cognitive science as a tool for characterizing psychological representations (Shepard, 1980). We did not collect similarity judgments over the full datasets as that is not feasible due to the quadratic growth in the number of required judgments as a function of the number of stimuli (e.g., 100 stimuli would require on the order 10,000 pairwise comparisons without including repetitions). In each trial of the task, participants were presented with a pair of stimuli (e.g., two videos) and were asked to rate how similar they were (“In each round you will be presented with two different videos and your task will be to simply judge how similar are the activities in them.”). Participants then responded on a 7‐item Likert scale ranging from 0 (“Completely Dissimilar”) to 6 (“Completely Similar”). Like the textual data, the similarity judgments for Mini‐Kinetics and Emotional Prosody were collected by Marjieh et al. (2023) and involved N=284 and N=252 participants, respectively. As for the similarity judgments on BOLD5K and WikiArt, these are newly collected and involved N=345 and N=343 participants, respectively. Demographic information is provided in Supplementary Table L1.

### 1.4 Data analysis

**Text Preprocessing**. We processed the textual data associated with each stimulus into a bag‐of‐words representation. For tags that were collected using the STEP‐Tag paradigm, we simply used the human evaluations to exclude all (22%) tags that satisfied one or more of the following criteria: (i) had an average quality score below three (out of five); (ii) had less than three evaluations; and (iii) were flagged at least twice. As for the free captions, here we applied traditional natural language processing techniques. First, we tokenized each caption using the WordPunctTokenizer method from the nltk Python package^3^ and removed stop words and tokens that contained nonalphabetic characters. We also corrected spelling mistakes using the pyspellchecker package.^4^ Next, parts of speech were determined for all tokens using the nltk.pos_tag method. Finally, we lemmatized all the remaining words using the WordNetLemmatizer method, which takes into account parts‐of‐speech and transforms them into shared forms, and then removed all duplications. For both types of data, the end result was a set of unique descriptors for each stimulus.

**Constructing Semantic Networks**. We followed a construction scheme that is similar to that of Steyvers and Tenenbaum (2005) to facilitate comparison. Specifically, in each stimulus domain, we defined an occurrence matrix $M_{ij}\in {\left\{0,1\right\}}^{\left|S|\times |T\right|}$, where S is the set of all stimuli in a given domain and T is the set of all unique tags. $M_{ij}=1$ if the tag $t_{j}$ appeared as a descriptor for stimulus $s_{i}$ and otherwise is zero. Then, a co‐occurrence matrix was constructed using the formula $C_{ij}=M^{⊤}M$, where ⊤ is the transpose operation. The co‐occurrence matrix $C_{ij}$ has the dimensions |T|×|T| (i.e., tag × tag), and it simply counts the number of times a pair of tags appeared as descriptors for the same stimulus. Using $C_{ij}$, we then constructed an undirected and unweighted graph G=(V,E) such that V=T, that is, each unique tag serves as a node, and $e_{ij}\in E$ whenever $C_{ij}>0$, that is, tags that co‐occurred are connected with an edge. To further ensure that the graph is connected and that all graph statistics are finite (see below), we restricted it to the largest connected component. In practice, we found that the size of the largest connected component encompassed more than 99% of the network for all datasets (in fact, it was 100% for all datasets except for the STEP‐Tag variants of BOLD5K and WikiArt, where it was 99.4% and 99.8%, respectively). When bootstrapping over stimuli, however, this number can be a bit smaller (since sampling with replacement ultimately results in repeated stimuli which do not add new unweighted edges because the tags already co‐occurred in the first stimulus). Nonetheless, we found that the average fraction of nodes in the largest connected component when bootstrapping over stimuli was bigger than 74% (see Supplementary Table E1 for additional details).

**Network Analysis**. All network analyses were performed using the networkx Python package.^5^ Average shortest path length and average clustering coefficient were computed using the methods average_shortest_path_length and average_clustering, respectively. As a reminder of the definitions from the literature (Steyvers and Tenenbaum, 2005), the average clustering coefficient is defined as the average of the node‐level clustering coefficient $C_{n}=2T_{n}/d_{n}\left(d_{n}-1\right)$, where $T_{n}$ is the number of node triangles going through node n, and $d_{n}$ is the node degree. Intuitively, this is the fraction of a node's neighbors who are themselves neighbors. In addition, we computed a common small‐worldness measure $\sigma =\left(C/C_{r}\right)/\left(L/L_{r}\right)$, which compares the clustering coefficient C and average shortest path L of a given graph against those of an equivalent random graph of the same size and average node degree $C_{r},L_{r}$ (Humphries, Gurney, & Prescott, 2006; Humphries & Gurney, 2008). A graph is said to be small‐world whenever σ>1. To construct the equivalent random graph, we used a networkx random graph generator gnp_random_graph with the same number of nodes n and an edge probability of $p=\bar{d}/n$, where $\bar{d}$ is the average degree in the original graph (hence, an average node degree of $∼np=\bar{d}$). We repeated this process five times (with the exception of WordNet due to its size, see below) and took the mean over $L_{r}$ and $C_{r}$ and then computed σ. Finally, we computed the degree assortativity coefficient $r_{d}$ using degree_assortativity_coefficient, which measures the extent to which nodes of similar degree tend to be connected by an edge (Newman, 2003). Mathematically, this is given by the formula $r_{d}=\sum_{j,k}jk\left(e_{jk}-q_{j}q_{k}\right)/\sigma_{q}^{2}$, where $e_{jk}$ is the fraction of edges that connect a node with degree j with a node with degree k, $q_{k}=\sum_{j}e_{jk}$ is the marginal, and $\sigma_{q}$ is the standard deviation of $q_{k}$. Intuitively, $r_{d}$ measures the correlation between the degree values of connected nodes. It satisfies $-1\leq r_{d}\leq 1$, where r=1 indicates perfect assortativity and r=−1 indicates perfect disassortativity (or perfect negative correlation). We bootstrapped all measures by sampling over stimuli with replacement with 50 repetitions (due to the size of the networks, the above computations are time‐intensive, which renders larger bootstraps with, e.g., 1000 repetitions, impractical). The bootstrapped measures were normalized relative to the size of each bootstrapped network.

**Community Detection**. To get a sense of the clustering organization of the semantic networks (sometimes referred to as the network's “community structure”), we used the Louvain community detection algorithm (Blondel, Guillaume, Lambiotte, & Lefebvre, 2008), implemented in the networkx package as louvain_communities with a resolution parameter of 1, a threshold parameter of $10^{-7}$, and unit edge weights. We used the Louvain algorithm due to computational efficiency considerations, as it is relatively fast for larger networks. This algorithm finds clusters by maximizing a measure of clustering quality known as modularity (Clauset, Newman, & Moore, 2004). The modularity score Q compares the number of within‐cluster links against the overall number of links that cluster members have. Formally, for undirected graphs, it is given by $Q=\sum_{c=1}^{n}\left[\frac{L_{c}}{m}-{\left(\frac{k_{c}}{2m}\right)}^{2}\right]$, where $L_{c}$ is the number of within‐cluster edges, m is the number of edges, n is the number of clusters, and $k_{c}$ is the sum of the degrees of the nodes within cluster c (also computed with networkx). The Louvain algorithm proceeds in two phases that are repeated iteratively. First, it assigns each node to its own cluster and then iteratively attempts to reassign each of these nodes to one of its neighbors' clusters such that the gain in modularity is maximized. The process repeats until there is no remaining gain. In the second phase, all nodes assigned to the same cluster are treated as a single node in a new graph, and the process of cluster reassignment repeats resulting in larger clusters. The phases keep alternating until there is no remaining gain (additional technical details can be found in Blondel et al. (2008)). This algorithm has been shown to outperform multiple popular clustering techniques and is widely used due to its efficiency (Blondel et al., 2008). As a control, in Supplementary Appendix F, we considered another popular clustering algorithm known as the Clauset–Newman–Moore (CNM; Clauset et al. (2004)) algorithm. This algorithm starts similarly to Louvain by assigning each node to its own cluster, but then attempts to maximize modularity by greedily merging pairs of clusters that lead to the largest increase in modularity until there are no additional gains. We found that CNM yielded correlated but generally lower quality solutions, suggesting that the Louvain algorithm was indeed a sensible choice (additional details are given in Supplementary Appendix F). The modularity scores using the Louvain algorithm ranged between .39−.51 for STEP‐Tag networks and somewhat lower but still positive for captions (.12−.21; individual values are provided in Supplementary Tables F1– F4). Overall, these values provide a good practical indication for significant community structure (Clauset et al., 2004). To estimate the stability of the resulting clustering, we ran the algorithm multiple times and then computed the consistency across all pairs of clusterings. This was done using the Adjusted Rand Index (ARI), which is a label‐free measure of clustering consistency (Hubert & Arabie, 1985; Rand, 1971). The Rand index is defined as R=(b+c)/a, where b is the number of pairs of tags that are grouped in the same community in the first and the second clustering, c is the number of pairs of tags that are grouped in different communities in both the first and second clusterings, and a is the total number of pairs. The ARI is the adjusted‐for‐chance Rand index, which we computed using the adjusted_rand_score method in the scikit‐learn Python package. An ARI of 0 is consistent with random clustering, an ARI of 1 indicates identical clustering (up to label permutations), and an ARI of −0.5 indicates discordant clusterings. Since the process is stochastic, we repeated it 1000 times, and computed confidence intervals. We report all ARIs in the Results section below. Finally, to find the most central nodes within each cluster, we simply computed the nodes' degree (i.e., number of edges) when restricted to their assigned cluster.

**Power‐law Analysis**. The evaluation of power‐law distributions requires special care due to the sensitivity of the distribution tails to statistical fluctuations, which can impact regression analyses (Alstott et al., 2014; Broido & Clauset, 2019; Clauset et al., 2009). As such, alternative methods based on maximum likelihood estimation have been proposed (Clauset et al., 2009), which we adopt here. We carry all such analyses using the powerlaw Python package (Alstott et al., 2014), which automates the process (i.e., model fitting, visualization, and statistical significance testing). In a nutshell, the process has two steps (Alstott et al., 2014): (i) Identifying the scaling range which corresponds to finding a minimum degree value $x_{min}$ above which the scaling relationship begins and the tail degree distribution is defined. This is done by fitting a power‐law distribution starting from each unique degree value in the data (via maximum likelihood) and then selecting the one that minimizes the Kolmogorov–Smirnov (KS) statistic between the empirical distribution and the model $D=\max_{x\geq x_{min}}\left|S\left(x\right)-P\left(x\right)\right|$, where S(x) and P(x) are the complementary cumulative distribution functions (CCDFs) of the fitted model and data, respectively (Clauset et al., 2009). (ii) Comparing the fitted power law ($x^{-\alpha }$) from the previous step against alternative candidate models using log‐likelihood tests (see Appendix C in Clauset et al. (2009) for technical details regarding *p*‐value computation). We consider four common alternatives: exponential ($e^{-\lambda x}$), stretched exponential (${\left(\lambda x\right)}^{\beta -1}e^{-{\left(\lambda x\right)}^{\beta }}$, 0<β<1), log‐normal ($x^{-1}e^{-{\left(\log x-\mu \right)}^{2}/2\sigma^{2}}$), and truncated power law ($x^{-\hat{\alpha }}e^{-\lambda x}$). The exponential is useful as it is not a heavy‐tailed distribution. The stretched exponential and log‐normal are useful because they are non‐power‐law heavy tailed distributions. Finally, the truncated power‐law distribution is informative because it accounts for cutoff effects that can lead to very large hubs in empirical distributions not being as common as one would expect from a perfect power law (e.g., due to finite size or growth bounds, Pastor‐Satorras & Vespignani (2002); see Discussion). Put differently, this distribution captures cases where there is an intermediate heavy‐tailed regime that is ultimately truncated due to network constraints. An additional power‐law plausibility test has also been proposed (Clauset et al., 2009), whereby a bootstrapping process is used to repeatedly sample data from the the fitted (perfect) power law and then iteratively fitting new power‐law models to such data and comparing their KS statistic relative to the original KS statistic computed on the raw data. However, as noted by Alstott et al. (2014), apart from being computationally expensive, this approach does not account for noise in the tail, and passing or failing such a test does not inform us of whether there is a more suitable alternative model or modification, which can be readily tested with the more efficient log‐likelihood test. Nonetheless, we include that test in Supplementary Table H5 and confirm that it is consistent with the conclusions of the log‐likelihood test.

**Network Visualization**. We visualized the multimodal networks using the gephi package (Bastian, Heymann, & Jacomy, 2009). For presentation purposes only, we pruned the networks by subselecting the top 1500 edges based on their weights. Moreover, we colored the nodes based on their community cluster (modularity class) as detected by a run of the Louvain community detection algorithm (Blondel et al., 2008).

### 1.5 Behavioral evaluations

**Predicting Similarity Judgments**. To evaluate the extent to which the semantic descriptors derived from each stimulus domain can capture the way participants directly perceive the stimuli in that domain (i.e., when they are not forced to describe the stimuli in words), we adopted a technique used in Marjieh et al. (2023). Specifically, given a stimulus i and its textual descriptors $t_{i}$ (tags or captions), we converted $t_{i}$ into a semantic vector $v_{i}$ using a suitable text embedding model (see Supplementary Appendix G for additional details). Intuitively, $v_{i}$ provides a quantitative representation of the semantic content of the descriptors $t_{i}$. Then, for each pair of stimuli i and j, we generated quantitative similarity predictions ${\hat{s}}_{ij}$ purely based on the text data by computing the cosine similarity between the two embedding vectors $v_{i}$ and $v_{j}$. Then, to quantify performance, we computed the Pearson correlation coefficient between the textual predictions ${\hat{s}}_{ij}$ and the human similarity matrices $s_{ij}$ for each domain (we focused on the upper triangular part of these matrices since they are symmetric with unit diagonal). This approach is akin to representational similarity analysis (Kriegeskorte, Mur, & Bandettini, 2008). We then compared the resulting correlation scores against human inter‐rater reliability (IRR), which we computed using a split‐half method with a Spearman–Brown correction (Brown, 1910) and 1000 repetitions. As an additional baseline, we repeated the process using domain‐specific models that can embed the stimuli directly into vector representations without any textual descriptors. In that case, each stimulus i is embedded directly into a vector $u_{i}$ and a prediction can be derived in the same way using cosine similarity. We ran the full suite of models described in Marjieh et al. (2023), which comprises 611 stimulus embedding models, in addition to text embedding models, and restricted our analysis to the best‐performing ones in each domain which we describe next (see Supplementary Appendix G for more technical details). For BOLD5K, the best performing models were: Vision Transformer (ViT; Dosovitskiy et al. (2020)), ConceptNet NumberBatch (CNNB; Speer, Chin, & Havasi (2017)), and SimCSE RoBERTa (Gao, Yao, & Chen, 2021) for images, tags, and captions, respectively. Next, for Mini‐Kinetics, the models were SlowFast (Feichtenhofer, Fan, Malik, & He, 2019), CNNB, and SimCSE RoBERTa, for videos, tags, and captions, respectively. As for WikiArt, the models were ConvNeXt (Liu et al., 2022), CNNB, and BERT (Devlin, Chang, Lee, & Toutanova, 2018), for images, tags, and captions, respectively. Finally, for Emotional Prosody, the models were Wav2Vec (Baevski, Zhou, Mohamed, & Auli, 2020), CNNB, and SimCSE RoBERTa, for audio, tags, and captions, respectively.

**Comparison to Lexical Semantic Networks**. As an additional baseline, we evaluated the extent to which our multimodal networks shared similar properties with lexical semantic networks. Since the original processed networks of Steyvers and Tenenbaum (2005) were not available, we reconstructed our version of the free association and WordNet networks based on publicly available source data and consistent procedures (i.e., using undirected edges, and restricting to the largest connected component). In particular, we accessed the free association data through Appendix A of Nelson et al. (2004), and the WordNet data through the following repository.^6^ The free association network contained 5019 words, and consistent with Steyvers and Tenenbaum (2005), we connected pairs of words if one word was provided at least twice as a free association response when the other was used as a cue. As for WordNet, the network contained 146,416 words and we simply used WordNet's available edge structure without modification.

**Reaction Time in Lexical Decision Tasks**. For a final evaluation, we checked whether node degree in the derived multimodal networks can predict reaction time in a lexical decision task. Here, we relied on the reaction time data of Balota et al. (2007). In this task, participants were presented with a string of letters and were asked to indicate if it corresponded to a real word or a nonword by pressing a button as a way of measuring their reaction time. We accessed the data through the following OSF repository.^7^ For the purpose of our analysis, we focused on the subset of trials that involved correct identification and extracted their reaction time. This comprised 2,349,471 judgments from 814 native English speakers. As a preprocessing step, we z‐scored the reaction times within participant and averaged them per word. For each network, we then computed the Spearman correlation ρ between the log degree and the average reaction time across shared words (we also provide Pearson correlations in Supplementary Appendix K). As an additional analysis, we also repeated the process after controlling for word frequency to see if there are any residual correlations that are not shared with frequency (these measures are naturally correlated; Steyvers and Tenenbaum (2005)). Word frequency was estimated using the wordfreq Python package,^8^ and the contribution of frequency was partialed out by fitting a linear regressor with log‐degree and log‐frequency as features and subtracting the contribution of log‐frequency.

### 1.6 Transparency and openness

All data and codes used in the present work are made available in the following OSF repository: https://osf.io/j9hva/overview. All participants provided informed consent prior to participation in accordance with a Princeton University Institutional Review Board protocol (10859) and a Max Planck Society Ethics Council protocol (2021_42).

## 2 Results

### 2.1 Structural analysis of semantic networks

**Fig. 3:**
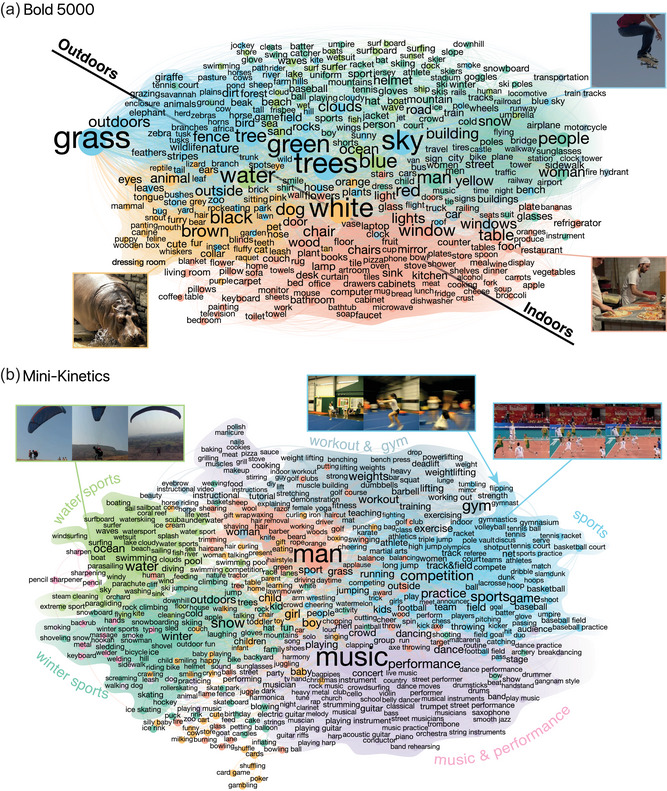
Multimodal semantic networks I. (A) BOLD5K images. (B) Mini‐Kinetics videos. Semantic clusters computed with Louvain community detection algorithm (see Methods) are marked with different colors. We added speculative community labels and axes based on our impression for the communities' content. *Note*. Sample images and video excerpts are reproduced from the datasets of Chang et al. (2019) and Xie et al. (2018), respectively.

**Fig. 4:**
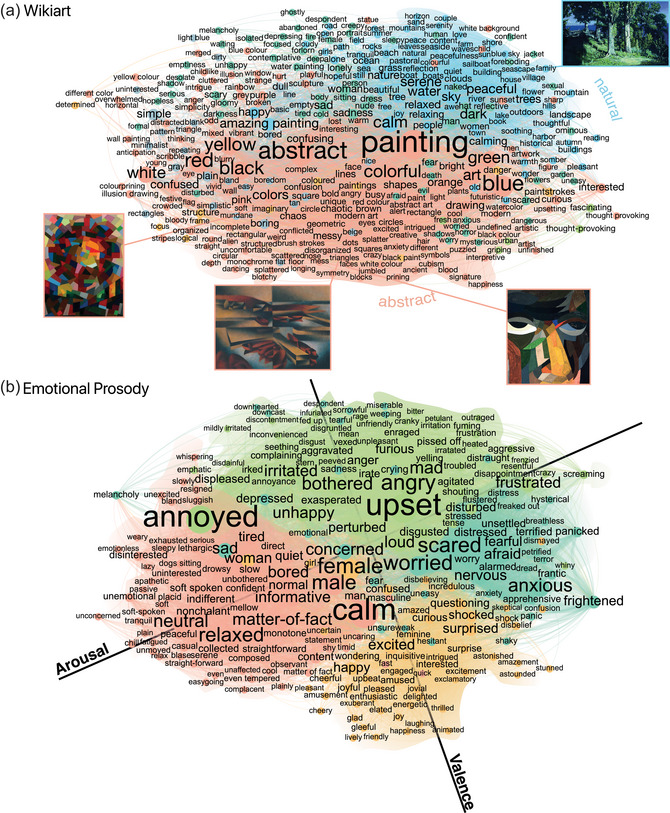
Multimodal semantic networks II. **(**A) WikiArt. (B) Emotional prosody recordings (speculative conceptual axes underlaid). *Note*. Sample artworks are reproduced from WikiArt under a public domain licence: Cypresses on a Seashore by Arkhyp Kuindzhi, 1887; Head (Self Portrait) by Otto Freundlich, 1923; Harbour on Bornholm by Arthur Segal, 1928; Rosace II by Otto Freundlich, 1941.

We begin by analyzing the structure of the multimodal semantic networks that we derived through the STEP‐Tag procedure. As a reminder, in this paradigm, participants observe stimuli and tag them with words, in addition to rating tags provided by other participants. The process repeats iteratively so that tags with low average ratings are pruned out (Fig. 2; see Methods). The final tags are then used to construct multimodal semantic networks by connecting words that co‐occur in response to the same stimulus with an edge (see Methods). The resulting multimodal semantic networks from the STEP‐Tag procedure are shown in Figs. 3 and 4. These networks are very rich semantically and possess interpretable semantic hubs (Table 1). In the case of BOLD5K (Fig. 3A), the network comprised 6751 unique words and the most connected were *white* (1290), *trees* (1244), *grass* (1211), *green* (1196), *sky* (1109), *man* (1063), *blue* (974), *water* (939), *people* (935), and *red* (913), which capture basic features of natural scenes. In addition to the hubs, the network has connected regions that follow an indoor‐outdoor broad decomposition. More concretely, we applied a Louvain community detection algorithm to analyze the different clusters (the algorithm found good clustering solutions across the different datasets as indexed by modularity scores in the range .39−.51; see Methods and Supplementary Appendix F for additional details). In this case, the algorithm detected 14 clusters (adjusted Rand index ARI=.57, 95% CIs = [.42,.71]; additional details are given in Supplementary Table F1). The largest cluster comprised 1308 nodes and the most connected words in it (based on within‐cluster degree, see Methods) were *table* (394), *window* (371), *chair* (338), and *lights* (326). The second largest cluster comprised 1238 nodes and the most connected words in it were *grass* (401), *man* (364), *sky* (338), and *green* (325). Finally, the third largest cluster comprised 1096 nodes and the most connected words in it were *white* (295), *trees* (294), *brown* (274), and *dog* (268).

**Table 1:** Top semantic hubs for the STEP‐Tag Networks

Dataset	Top hub words	Top hub degrees
BOLD5K	White, trees, grass, green, sky, man, blue, water, people, red	1290, 1244, 1211, 1196, 1109, 1063, 974, 939, 935, 913
Mini‐Kinetics	Man, music, woman, girl, competition, water, boy, snow, sports, gym	481, 452, 255, 235, 202, 195, 192, 188, 186, 183
WikiArt	Painting, abstract, blue, red, black, art, dark, colorful, calm, white	527, 463, 336, 316, 305, 295, 287, 269, 265, 252
Prosody	Calm, annoyed, upset, matter‐of‐fact, male, female, relaxed, worried, angry, loud	270, 261, 255, 206, 191, 188, 181, 178, 178, 164

As for Mini‐Kinetics (Fig. 3B), the network comprised 2181 unique words and the most connected words were *man* (481), *music* (452), *woman* (255), *girl* (235), *competition* (202), *water* (195), *boy* (192), *snow* (188), *sports* (186), and *gym* (183), which are intuitive in the context of human social activity. More broadly, the network covers a range of thematic regions, such as different forms of sports and musical engagement. Applying clustering analysis in this case yielded 13 clusters (ARI=.61, 95% CIs = [.50,.72]). The largest cluster comprised 344 nodes and the leading words were *music* (223), *performance* (113), *dance* (67), and *stage* (65). Next, the second largest cluster comprised 297 nodes and the top words were *baby* (90), *child* (85), *girl* (81), and *kid* (52). As for the third largest cluster, it comprised 258 nodes and the leading words were *sports* (105), *game* (99), *competition* (92), and *field* (66).

As for WikiArt (Fig. 4A), the semantic organization of the 2124 mined words is particularly interesting with low‐level regions associated with basic colors that, in turn, extend outward to moods and associations with varying degrees of valence and arousal. Indeed, the most connected words in this case were *painting* (527), *abstract* (463), *blue* (336), *red* (316), *black* (305), *art* (295), *dark* (287), *colorful* (269), *calm* (265), and *white* (252). The clustering algorithm detected 15 clusters (ARI=.33, 95% CIs = [.23,.44]). The leading cluster comprised 372 nodes, with the top words being *red* (114), *colorful* (103), *white* (97), and *yellow* (87). The second largest cluster contained 369 nodes with the leading words being *calm* (154), *water* (123), *serene* (118), and *peaceful* (116). As for the third largest cluster, it contained 355 nodes with the leading words being *abstract* (194), *black* (90), *messy* (63), and *confusing* (61).

Finally, for the Emotional Prosody dataset (Fig. 4B), the network comprised 787 unique words and has a strong valence‐arousal decomposition as expected from emotion research (Nook, Sasse, Lambert, McLaughlin, & Somerville, 2017; Russell, 1980) with semantic hubs at *calm* (270), *annoyed* (261), *upset* (255), *matter‐of‐fact* (206), *male* (191), *female* (188), *relaxed* (181), *worried* (178), *angry* (178), and *loud* (164). Applying clustering analysis, we found four clusters (ARI=.89, 95% CIs = [.71,.97]). The leading cluster contained 238 nodes and the leading words were *calm* (175), *relaxed* (120), *bored* (120), and *neutral* (118). The second cluster comprised 198 nodes and here the leading words were *happy* (103), *excited* (91), *surprised* (84), and *amused* (65). As for the third cluster, it contained 184 nodes, with the leading words being *annoyed* (138), *angry* (124), *mad* (93), and *furious* (93). For completeness, we also describe the fourth cluster: it comprised 167 nodes, and the leading words were *upset* (102), *worried* (101), *scared* (100), and *anxious* (87).

We remind the reader that the ARI is a label‐free measure for comparing pairs of clustering solutions (Hubert & Arabie, 1985; Rand, 1971). It is 0 for random solutions, 1 for perfectly aligned solutions, and approaches –0.5 for discordant solutions (i.e., if two nodes are in the same cluster in one solution, then they are in different clusters in the other solution). An ARI value that is significantly above 0 indicates systematic overlap. Values in the range .57 – .89 indicate good agreement, considering the stochastic nature of the Louvain algorithm and the size of the networks. The WikiArt clustering solution was somewhat more variable with an ARI of .33. This may be due to the highly subjective nature of the domain involving abstract art and textures that can be difficult to describe in simple terms.

We next turn to a finer analysis of the statistical graph properties of the multimodal networks. In particular, we wanted to evaluate whether these networks had small‐world properties. As noted earlier, small‐world networks are characterized by sparse connectivity, short average path length, and high local clustering. We summarize these metrics for each of the datasets in Table 2 (see Methods). We found that in all four cases the semantic networks were very sparse meaning that most words co‐occurred with only a small fraction of the full lexicon (<3.5%). Despite this sparsity, the average path length between two words did not exceed three steps for all conditions, and the average clustering coefficient was very high (>.7) (i.e., neighbors of a given node were themselves likely to be neighbors with an average probability that exceeds .7). This is already indicative of small‐world behavior, but to assess this more rigorously, we computed the expected average shortest path length and clustering for equivalent random graphs, and then compared them against our data using the small‐worldness coefficient σ (see Methods). In all cases, we found that σ≫1 (between 20.5 and 160.8, see Table 2), confirming our expectation that the semantic networks indeed admit a small‐world topology. Finally, we computed the degree assortativity coefficient for each network which captures the extent to which nodes of similar degree tend to connect (see Methods). Here, we found that all networks exhibited negative degree assortativity (Table 2), meaning that lower degree nodes tended to connect to higher degree nodes.

**Table 2:** Graph statistics for the multimodal semantic networks and their 95% confidence intervals

Dataset	$\bar{d}$	${\text{CI}}_{\bar{d}}$	$\bar{s}$	${\text{CI}}_{\bar{s}}$	L	${\text{CI}}_{L}$
BOLD5K	26.3	[25.8, 26.8]	0.50%	[0.49, 0.52]%	2.77	[2.75, 2.78]
Mini‐Kinetics	14.7	[14.3, 15.2]	0.87%	[0.84, 0.90]%	3.02	[2.97, 3.05]
WikiArt	17.4	[16.9, 17.9]	1.10%	[1.06, 1.15]%	2.80	[2.76, 2.84]
Prosody	22.6	[21.8, 23.2]	3.44%	[3.24, 3.60]%	2.49	[2.46, 2.51]

Dataset	C	${\text{CI}}_{C}$	σ	${\text{CI}}_{\sigma }$	$r_{d}$	${\text{CI}}_{r_{d}}$
BOLD5K	.771	[.768, .776]	160.8	[155.6, 166.2]	−0.133	[−0.137, −0.130]
Mini‐Kinetics	.762	[.752, .769]	88.6	[82.4, 94.9]	−0.062	[−0.069, −0.056]
WikiArt	.780	[.773, .789]	72.1	[68.3, 75.8]	−0.093	[−0.101, −0.085]
Prosody	.727	[.716, .735]	20.5	[19.4, 22.1]	−0.170	[−0.181, −0.160]

*Note*. The measures are: average node degree $\bar{d}$, average sparsity $\bar{s}=\bar{d}/\left|G\right|$, where |G| is the size of the network, L is the average shortest path length, C is the average clustering coefficient, σ is the small‐worldness coefficient, and $r_{d}$ is the degree assortativity. CI indicates 95% confidence intervals. See Methods for full details. Additional metrics are provided in Supplementary Table E1.

As an additional control, we wanted to ensure that the observed regularities were not simply an artifact of our STEP‐Tag semantic mining approach. To that end, we repeated the network analysis for each stimulus dataset based on free captions of the stimuli (see Methods). The resulting network statistics are provided in Supplementary Table D1. As with STEP‐Tag networks, we found that the caption‐based networks were characterized by short average path length (L<2.3 across datasets), high sparsity ($\bar{s}<4.2\%$), high clustering (C>.76), large small‐worldness coefficient (σ>19.7), and negative degree assortativity ($r_{d}<-.23$).

### 2.2 Power‐law scaling evaluation

Next, to evaluate whether the multimodal semantic networks are scale‐free, we used a standard maximum likelihood technique for evaluating power‐law distributions (Alstott et al., 2014; Clauset et al., 2009, see Methods). In addition to the power‐law model, we consider four alternative distributions, namely, exponential, stretched exponential, log‐normal, and truncated power law. The exponential provides a non‐heavy‐tailed candidate, while the stretched exponential and log‐normal distributions provide non‐power‐law heavy‐tailed alternatives. Finally, the truncated power law captures the impact of cutoff effects on power‐law distributions, whereby very large hubs cannot be as common as one would expect from a perfect power‐law relation. This results in an intermediate heavy‐tailed regime that is ultimately truncated due to growth constraints (see Discussion).

**Fig. 5:**
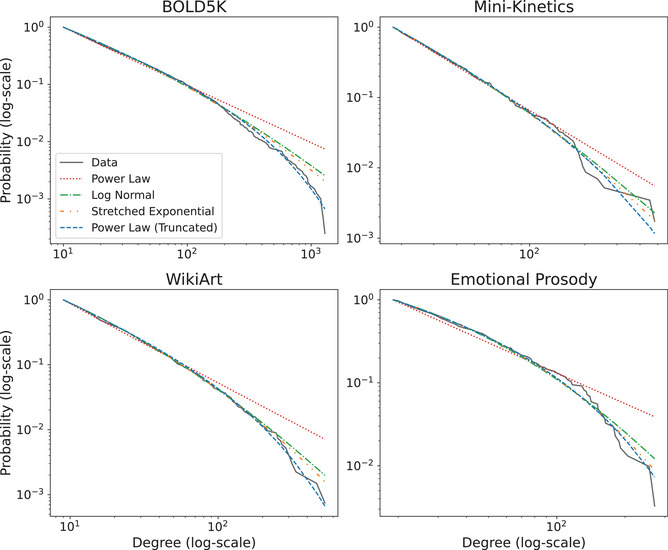
Complementary cumulative distribution function of the tail degree distribution (see Methods) for all STEP‐Tag datasets on a log‐log scale, along with different fitted models (exponential model provided separately in Supplementary Fig. C1 due to poor fit).

The empirical CCDFs, as well as the fitted models, are shown in Fig. 5 on a log‐log scale (with the exception of the exponential model which yielded a poor fit relative to the other models and so we provide it separately in Supplementary Fig. C1; raw degree distributions are shown in Supplementary Fig. C2). Qualitatively, we found that the CCDFs appear to exhibit intermediate heavy tails with large‐scale cutoffs. Quantitatively, we provide the results of log‐likelihood tests for the truncated power law against all other models in Table 3, in addition to a comparison between the power law and exponential models (additional power‐law comparisons are provided in Supplementary Table H1; see Methods). We found that the truncated power law significantly out‐performed all competing models in the BOLD5K and Emotional Prosody datasets, and that it could not be ruled out by the other models in Mini‐Kinetics and WikiArt (the truncated power‐law outperformed all models as indicated by a positive log‐likelihood test but the result was significant only against the power law and exponential models; for semantic networks based on free captions, the truncated power law outperformed in all conditions; see below and Supplementary Table H2). The truncated power law also provided good fit to the distribution tail as quantified by the mean and 95% CIs of the explained variance: BOLD5K: .991 [.984,.997], Mini‐Kinetics: .969 [.952,.992], WikiArt: .958 [.399,.997], and Emotional Prosody: .972 [.911,.994] (confidence intervals were estimated by sampling from the degree distribution with replacement 1000 times). Additional metrics are provided in Supplementary Table H3. As for the power‐law exponents $\hat{\alpha }$ derived from the truncated power law, these were 1.85 (95% CI: [1.79,1.91]) for BOLD5K, 2.3 (95% CI: [1.92,2.60]) for Mini‐Kinetics, 2.01 (95% CI: [1.00,2.44]) for WikiArt, and 1.49 (95% CIs: [1.00,1.97]) for Emotional Prosody (see additional parameters in Supplementary Table H4).

**Table 3:** Log‐likelihood ratio tests for the STEP‐Tag degree distributions

Dataset	Base	Alternative	LLR	p	Conclusion
BOLD5K	Power Law	Exponential	1368.836	.0000	Power Law
BOLD5K	Power Law (Tr.)	Exponential	1408.027	.0000	Power Law (Tr.)
BOLD5K	Power Law (Tr.)	Log‐Normal	12.439	.0000	Power Law (Tr.)
BOLD5K	Power Law (Tr.)	Stretched Exp.	9.962	.0000	Power Law (Tr.)
BOLD5K	Power Law (Tr.)	Power Law	39.191	.0000	Power Law (Tr.)
Mini‐Kinetics	Power Law	Exponential	76.443	.0001	Power Law
Mini‐Kinetics	Power Law (Tr.)	Exponential	79.470	.0000	Power Law (Tr.)
Mini‐Kinetics	Power Law (Tr.)	Log‐Normal	0.799	.1653	Inconclusive
Mini‐Kinetics	Power Law (Tr.)	Stretched Exp.	0.676	.2353	Inconclusive
Mini‐Kinetics	Power Law (Tr.)	Power Law	3.028	.0139	Power Law (Tr.)
WikiArt	Power Law	Exponential	268.948	.0000	Power Law
WikiArt	Power Law (Tr.)	Exponential	282.432	.0000	Power Law (Tr.)
WikiArt	Power Law (Tr.)	Log‐Normal	2.022	.1412	Inconclusive
WikiArt	Power Law (Tr.)	Stretched Exp.	1.435	.2174	Inconclusive
WikiArt	Power Law (Tr.)	Power Law	13.484	.0000	Power Law (Tr.)
Prosody	Power Law	Exponential	0.748	.9313	Inconclusive
Prosody	Power Law (Tr.)	Exponential	15.068	.0034	Power Law (Tr.)
Prosody	Power Law (Tr.)	Log‐Normal	2.333	.0054	Power Law (Tr.)
Prosody	Power Law (Tr.)	Stretched Exp.	1.048	.0269	Power Law (Tr.)
Prosody	Power Law (Tr.)	Power Law	14.320	.0000	Power Law (Tr.)

*Note*. Tr. indicates truncation (see Methods). LLR is the log‐likelihood ratio test (positive value favors base model). Suitable nested log‐likelihood tests were used when one of the functions was a subset of the other (e.g., when comparing a truncated power law with a regular power law; Alstott et al. (2014)). p indicates the *p*‐value of the test. Significance was determined with a p<.05 threshold. A value of .0000 indicates p<.0001. See Supplementary Table H1 for additional comparisons.

As with the structural analysis, we repeated the power‐law evaluation process for the semantic networks derived from free captions (see Methods). The resulting tail CCDFs and fitting statistics are provided in Supplementary Fig. D1 and Supplementary Tables H2 and H3, respectively. Our findings in this case were consistent, namely, that the truncated power‐law model significantly outperformed all competing models as determined by the log‐likelihood ratio test (Supplementary Table H2), and that it yielded good fit to the tail distribution (variance explained 95% CIs: BOLD5K: [.981,.998], Mini‐Kinetics [.982,.998], WikiArt [.972,.998], Emotional Prosody: [.972,.998]). Likewise, the derived exponents spanned a similar global range (95% CIs for $\hat{\alpha }$: BOLD5K: [1.60,1.96], Mini‐Kinetics: [1.75,1.86], WikiArt: [1.57,3.04], Emotional Prosody: [1.00,2.42]; additional parameters are provided in Supplementary Table H4).

### 2.3 Behavioral evaluation I: Predicting human similarity judgments

**Fig. 6:**
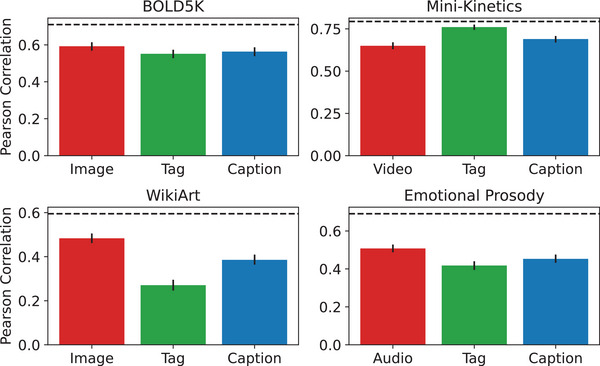
Correlation with human similarity judgments for the best embedding model in each input category (see Methods). Similarity predictions were generated by embedding each input type using either a text‐based or a domain‐specific deep model and then generating a similarity prediction using cosine similarity which was then correlated against human data. Error bars indicate 95% confidence intervals. Dashed horizontal line indicates human inter‐rater reliability. Correlation matrices between predictors are provided in Supplementary Table G1.

Moving beyond the statistical graph properties, we next evaluated the extent to which our semantic descriptors were indeed predictive of the way humans perceive the stimuli. To do that, we used similarity judgments as a proxy for sensory experiences where humans perceive the stimuli directly and compare them (Marjieh et al., 2023), and then evaluated how well those judgments can be predicted based on text embeddings of the descriptors in each domain (see Methods). We then compared their performance against that of a suit of deep embedding models applied to the stimuli directly as a proxy for a predictor that is not language‐mediated (see Methods). Each similarity set comprised 100 stimuli, and 100×99/2=4950 unique pairs, each with their average similarity score (the number of judgments per domain is provided in Supplementary Table M3; rating distribution statistics are provided in Supplementary Table M4). The results of this analysis are summarized in Fig. 6. We found that across all domains, the textual descriptors yielded significant correlations. For BOLD5K, the correlation confidence intervals (CIs) were [.57,.61], [.53,.57], [.54,.59] for images, tags, and captions, respectively, and the IRR CI was [.70,.72]. Next, for Mini‐Kinetics, we have [.63,.67], [.74,.78], [.67,.71] for video, tags, and captions, respectively, and [.79,.80] for the IRR. Interestingly, the tag‐based embeddings yielded in this case a correlation that is nearly as high as the IRR. Next, for WikiArt, we have [.46,.51], [.25,.30], [.36,.41] for images, tags, and captions, respectively, and for the IRR CI, we have [.58,.61]. Here, we see that even though all embeddings are significantly correlated, the image‐based embeddings had the highest correlation, whereas the tags had the lowest. One possible explanation for this is that the WikiArt domain contains abstract art that is characterized by low‐level features such as texture and color shades that are easy to perceive visually but can be hard to condense into concise tags. Finally, for the emotional prosody domain, we have [.49,.53], [.39,.44], [.43,.48] for audio, tags, and captions, respectively, and [.68,.70] for the IRR. We note that similar to Marjieh et al. (2023), a gap between the IRR and the unimodal approaches is expected as humans integrate both semantic and low‐level information (e.g., color shades and texture) when forming a similarity judgment. This could be accounted for by constructing multimodal stacked representations (Marjieh et al., 2023); however, for the purpose of studying the large‐scale structure of semantic networks, this analysis is tangential.

### 2.4 Behavioral evaluation II: Comparison to lexical semantic networks

Next, we wanted to evaluate whether the derived multimodal networks shared similar features with the lexical networks studied in Steyvers and Tenenbaum (2005). Since the processed networks of Steyvers and Tenenbaum (2005) were not available, we constructed our own version of their free association and WordNet networks based on available source data (see Methods). The network statistics are provided in Table 4. Apart from replicating the finding that those lexical networks are indeed small‐world as captured by the large σ coefficients (Free Associations: 42.3, WordNet: 2038.9), we also found that they exhibit disassortative mixing by degree with negative assortativity coefficients that are comparable to those of the multimodal networks (Free Associations: −.128, WordNet: −.093; compare to Tables 2 and D1).

**Table 4:** Graph statistics for two lexical semantic networks: Free Associations and WordNet

Dataset	$\bar{d}$	$\bar{s}$	L	C	σ	$r_{d}$
Associations	22.0	0.44%	3.04	.186	42.3	−0.128
WordNet	7.5	0.01%	5.49	.074	2038.9	−0.093

*Note*. The measures are: average node degree $\bar{d}$, average sparsity $\bar{s}=\bar{d}/\left|G\right|$, where |G| is the size of the network, L is the average shortest path length, C is the average clustering coefficient, σ is the small‐worldness coefficient, and $r_{d}$ is the degree assortativity coefficient. Note that 95% CIs are not available in this case because there are no underlying stimuli to bootstrap over.

Likewise, we subjected the lexical networks to the same power‐law evaluation analysis by fitting different models using maximum likelihood estimation and applying log‐likelihood ratio tests (see Supplementary Tables I1 and I2 and Supplementary Fig. I1). Interestingly, we found that the truncated power law again performed best, though the pure power law in this case could not be ruled out by the competing heavy‐tailed non‐power‐law models (we emphasize that the likelihood test used takes into account the fact that the truncated power law and the regular power law are nested; Alstott et al. (2014)). As for the fitted truncated power‐law exponents $\hat{\alpha }$, these were 2.85 for free associations, and 2.29 for WordNet (additional metrics are provided in Supplementary Table I3). These exponents are larger than those derived from the multimodal semantic networks earlier (the average exponent value in that case was 1.87), although they are also somewhat smaller than those derived by Steyvers and Tenenbaum (2005), which were ∼3. Note, however, that those latter exponents were derived by applying linear regression to the empirical degree distributions, which can lead to inaccuracies in estimation (Clauset et al., 2009).

Finally, we evaluated whether the semantic and lexical networks had mutual semantic hubs or themes (i.e., words with high network degree). To that end, we computed the overlap between the top 100 word hubs (in terms of node degree) for each multimodal and lexical network and then computed the overlap. For the free association network, we found that the overlap with the STEP‐Tag networks in descending order was: BOLD5K (22%), Mini‐Kinetics (15%), WikiArt (15%), and Emotional Prosody (5%) (the shared words are provided in Supplementary Appendix J). Likewise, the overlap with the Free Caption networks was: BOLD5K (24%), Mini‐Kinetics (24%), WikiArt (23%), and Emotional Prosody (8%). Overall, we see that BOLD5K provided the largest overlap, which highlights the role of natural scenes in the organization of human associative networks. As for WordNet, the overlap was much lower (≤3% across datasets for STEP‐Tag, and ≤13% for Captions). This may reflect the fact that WordNet is a synthetically constructed network that contains large technical categories (e.g., “jurisprudence” and “asteraceae”). To get a finer sense of the thematic overlap with the free association network, we also checked whether the multimodal and free association networks shared within‐community themes, which we operationalized as shared nodes that appeared within the top 20 nodes of their respective Louvain communities. We found that all datasets yielded meaningful overlapping themes. These included among other aquatic themes (water, ocean, fish, sea), animal themes (animal, bird, dog, furry), music themes (music, instrument, singer, song), food (food, fruit, bread), youth (girl, child, baby), and transportation (car, travel, road). Additional examples are provided in Supplementary Appendix J.

### 2.5 Behavioral evaluation III: Predicting reaction time in lexical decision tasks

As a final evaluation, we tested whether node degree in the multimodal networks is correlated with reaction time in a lexical decision task (see Methods). In this task, participants were asked to judge whether string sequences corresponded to a real world or a nonword by pressing a button (Balota et al., 2007). The results for the multimodal and lexical networks are provided in Table 5. We found that all networks, both lexical and multimodal, yielded significant negative correlations. This means that nodes with higher network degree tend to be correctly identified more quickly in the lexical decision task. Among the multimodal networks, BOLD5K provided the highest correlation in both the STEP‐Tag (−.348, $p<10^{-4}$) and Free Caption (−.419, $p<10^{-4}$) categories. These correlations were comparable to those derived from the lexical networks (though slightly lower than the free association network). Interestingly, after partialing out the effect of word frequency (see Methods) to see if node degree captures distinct variance (since these are naturally correlated; Steyvers and Tenenbaum (2005)), we found that only Free Associations and BOLD5K exhibited significant and non‐negligible residual correlations (though small as in Steyvers and Tenenbaum (2005)). These were −.135 ($p<10^{-4}$) for BOLD5K (Tags), −.151 ($p<10^{-4}$) for BOLD5K (Captions), and −.246 ($p<10^{-4}$) for free associations (see Supplementary Table K2 for additional details). As with the hub overlap analysis earlier, these results highlight the relationship between natural scenes and semantic organization.

**Table 5:** Correlation between network log‐degree and reaction time in a lexical decision task

Dataset	$N_{shared}$	ρ	p
BOLD5K (Tags)	3741	−0.348	$<10^{-4}$
Mini‐Kinetics (Tags)	1383	−0.183	$<10^{-4}$
WikiArt (Tags)	1750	−0.203	$<10^{-4}$
Prosody (Tags)	624	−0.156	$<10^{-4}$
BOLD5K (Captions)	5767	−0.419	$<10^{-4}$
Mini‐Kinetics (Captions)	3325	−0.365	$<10^{-4}$
WikiArt (Captions)	5492	−0.357	$<10^{-4}$
Prosody (Captions)	2385	−0.255	$<10^{-4}$
Associations	4913	−0.490	$<10^{-4}$
WordNet	27,990	−0.376	$<10^{-4}$

*Note*. The measures are: the number of shared words between the network and the lexical decision task $N_{shared}$, Spearman correlation ρ between log‐degree and reaction time, and the corresponding *p*‐value p (Pearson correlations are provided in Supplementary Table K1).

## 3 Discussion

Our results provide clear evidence from diverse domains, modalities, and behavioral datasets that multimodal semantic networks, that is, as derived from directly labeling sensory stimuli, admit a small‐world organization with a truncated power‐law degree distribution (sometimes referred to as scale‐free with a cutoff). Moreover, upon a reanalysis of two classic lexical networks based on free associations and WordNet, we show that these too share the same characteristics. We also show that the multimodal networks are predictive of behavioral data such as direct similarity judgments and reaction times, further supporting their behavioral relevance.

These findings extend and inform previous work on semantic networks. Finding that multimodal networks that emerge from labeling diverse naturalistic domains exhibit a small‐world structure is consistent with prior work on lexical networks such as that of Steyvers and Tenenbaum (2005) and others. However, it adds a new insight, namely, that this property, which is usually noted for its support of search efficiency (Borge‐Holthoefer and Arenas, 2010), is also compatible with the regularities of the environment (e.g., as seen from the natural images and video datasets; Fig. 3), echoing the classic literature on small‐world networks (Watts & Strogatz, 1998). Interestingly, our results also highlight that the exact way in which this structural organization is manifested differs substantially between domains, and may be shaped by other large‐scale considerations such as indoor‐outdoor distinctions for natural images (Fig. 3A) and valence‐arousal dimensions in the case of emotional prosody (Fig. 4B). Moreover, the dominance of basic color concepts in more than one dataset (Figs. 3A and 4A) suggests that certain core concepts may play a central organizing role that extends across multiple modalities.

Likewise, finding that multimodal and lexical networks are better captured by a truncated power law generalizes prior statements in the literature (e.g., Morais, Olsson, & Schooler, 2013; Utsumi, 2015) to a new class of multimodal networks. Truncated power‐law distributions imply that although most nodes have a small number of connections and a few hubs have many, the frequency of those hubs is not as high as one would expect from a perfect power law. This could be because there are some constraints on the ability of words to grow in connectivity; however, there is no simple way of determining which words ought to have been more connected a priori and we leave this to future work. Note that this truncation may arise from methodological constraints, for example, from our convergence criterion for the STEP‐Tag chains (see Methods). However, the fact that this truncation is observed repeatedly across different data collection approaches (STEP‐Tag and free captions), as well as in prior lexical networks, suggests that there may be other, possibly cognitive, constraints at play. Future work could investigate the nature of such constraints and the way they impact growth (see, e.g., Utsumi, 2015 for one example).

Relatedly, the fact that our derived multimodal networks exhibited small‐world and truncated power‐law properties based on two rather different semantic mining techniques (STEP‐Tag vs. free captions) further strengthens the idea that these reflect the semantic regularities of naturalistic datasets. However, it is worth noting that the specific network statistics elicited from the two techniques differ (Tables 2 and D1). This is to be expected, as the two techniques have different emphases. STEP‐Tag aims for succinct descriptors that are agreed on by multiple individuals due to the rating‐and‐pruning mechanism (Fig. 2A). Captions, on the other hand, allow each participant to freely describe the stimulus, preserving individual variation. The difference between the two techniques can, therefore, be thought of effectively as the difference between the intersection and union of the descriptors provided by participants. Nonetheless, we reiterate that both techniques demonstrated the small‐worldness and truncated power‐law properties found in lexical networks, which is the main contribution of the present work.

In terms of fitted (truncated) power‐law exponents, we found that multimodal networks generally resulted in smaller values (1.5−2.3) than their lexical counterparts (2.3−2.9), meaning that they are more densely connected. This is also consistent with the average shortest path length and clustering coefficient values, which for the multimodal networks varied in the range 2.0−3.0 and .73−.82, respectively, as opposed to 3.0−5.5 and .07−.19 for the lexical networks. This is also in line with prior reported lexical network statistics as in Borge‐Holthoefer and Arenas (2010), at least for the clustering and path length measures, as the modern likelihood‐based approach of Clauset et al. (2009) for evaluating power‐law distributions may not have been readily available at that point. This observation suggests that the internalized lexical networks are somewhat more conservative in their connectivity relative to the multimodal semantic networks. One possibility is that some connecting concepts are absent from the curated lexical datasets or those derived from free association but are preserved in the multimodal case which does not rely on memory. It would be informative to understand the origin of this difference and how it fits within a unified statistical learning framework such as that of Griffiths et al. (2007).

Beyond the statistical network properties, we also found that the multimodal networks shared thematic themes with the free association network (especially in the case of the BOLD5K dataset of natural images). This was reflected both at the broader level of comparing the most connected words (which does not rely on any community detection algorithms), as well as at the finer cluster level, which we detected using the Louvain algorithm. Note that in Supplementary Appendix F, we also considered another popular clustering algorithm (Clauset–Newman–Moore; Clauset et al. (2004)), which yielded some degree of correlation with the Louvain clusters but also some differences. However, it generally underperformed the Louvain algorithm in solution quality (as indicated by a lower modularity score across all datasets, as well as the tendency to cluster a small number of nodes [≤5] into their own cluster, which increased the overall number of detected clusters; compare Supplementary Tables F1 vs. F3 and F2 vs. F4). This suggests that the Louvain algorithm was a sensible choice. Overall, finding thematic overlap both without and with clustering further highlights the idea that associative networks may reflect the semantic regularities of the environment.

Although there is no simple equivalent for a growth mechanism in the case of multimodal semantic networks given that they reflect the structure of an external domain (e.g., natural scenes), our results support models of semantic growth such as preferential acquisition and lure of the associates (Hills et al., 2009) that integrate the structure of the external learning environment in the development process. In these models, internal semantic networks develop by acquiring words from an external semantic network that constitutes the learning environment (e.g., by acquiring the most connected word in the external network in the case of preferential acquisition, or the word that is connected to the largest number of already acquired words in the case of lure of the associates). This differs from the preferential attachment model used by Steyvers and Tenenbaum (2005), where the probability that a new word attaches to an old word depends only on the latter's degree in the internal network (see Fig. 5 in Hills et al. (2009) for an illustration). The external semantic network in these models is typically modeled on semantic networks that are derived from free associations (Nelson et al., 2004) but one may wonder how such networks are manifested in daily experience. Our multimodal networks suggest that merely by describing and labeling natural scenes and daily activities, humans can generate semantic networks with similar properties. Future work could explore the implications of this idea more closely by using multimodal networks as models of the external environment in tandem with suitable longitudinal data as in Hills et al. (2009) to test different mechanisms of semantic acquisition.

Finding that our multimodal networks are predictive of independent psychological measures such as similarity judgments and reaction times is analogous to other recent work on lexical semantic networks (e.g., De Deyne et al., 2019). However, it is important to note that the emphasis is somewhat different as these prior studies directly compare lexical networks (as in free associations) against other lexical judgments (e.g., word similarity) as a way of tapping into lexical processing. This differs from our case where we consider the compatibility of psychological data across different modalities (e.g., textual similarity vs. direct similarity judgments over images). The significance of our findings is in that they validate the psychological relevance of multimodal networks as useful representations of the way people perceive naturalistic stimuli and their evoked semantics.

As for the measures used for predicting similarity data based on embeddings of stimuli, tags, and captions, we found that the best performing measure depended on the domain (Fig. 6), which suggests that there is no intrinsic advantage for any one of these measures (e.g., due to variation in the amount of embedded data). For instance, for the video dataset (Mini‐Kinetics), the tags performed best, whereas for WikiArt, image embeddings outperformed. This highlights the complementary strengths of these measures: tags elicited from STEP‐Tag focus on succinct descriptors that are agreed on by multiple participants, whereas captions allow for individual variation by having people freely describe the stimuli. Direct stimulus embeddings, on the other hand, can capture low‐level features that are easy to perceive but hard to describe in words (such as textures and abstract shapes as found in WikiArt). It is also worth noting that the STEP‐Tag and caption methods are relatively calibrated in terms of participant effort time. These data were collected and analyzed by Marjieh et al. (2023) for the video and audio datasets, where the median participant time per stimulus was shown to be comparable (Video: 264 and 291 s for STEP‐Tag and captions, respectively; Audio: 230 and 187 s for STEP‐Tag and captions, respectively).

Finally, it is worth clarifying that our distinction between multimodal and lexical semantic networks is different from the distinction between “grounded” and “ungrounded” representations as used in the literatures on distributed semantics and large language models (e.g., Lewis, Zettersten, & Lupyan, 2019; Patel & Pavlick, 2022). Unlike the latter where the ungrounded representation is learned in the absence of any sensory experience, our lexical and multimodal networks both arise from agents who had extensive experience of the sensory world. Finding that these distinct network types nonetheless share consistent properties further strengthens this point.

We end by discussing some limitations of the present work, which point toward future research directions. First, our behavioral evaluation based on similarity judgments relied on text embeddings as they provide an effective way for quantifying semantic similarity. However, one could also use the derived network structure explicitly to construct similarity predictions, for example, through a mechanism of semantic activation on the network (De Deyne & Storms, 2008). Future work could look into how such an approach compares to the one based on text embeddings. In particular, it would be informative to see how it performs on domains like abstract art in which there was a sizable gap between the predictions based on image embeddings versus text embeddings. We should also note that the average split‐half IRR values for the similarity judgments ranged between .60 and .79 across domains. While these are not very high in absolute terms, they are substantial and significant given the complexity of the stimuli and the different aspects of similarity that they could capture, which may vary across individuals. Moreover, due to the quadratic scaling in the number of judgments needed to cover all pairs in a set of stimuli, the similarity experiments were quite substantial in terms of human data, each encompassing more than 20,000 human judgments per domain, and together covering more than 100,000 human judgments. Future work could consider even larger behavioral datasets and evaluate how far the IRR can be increased.

Second, similar to Steyvers and Tenenbaum (2005), we flattened the weights of the co‐occurrence matrices for compatibility with prior research and because we were primarily interested in the large‐scale undirected structure of the semantic networks. Note that the weights in free association networks encode directionality as one word is elicited in response to another. This differs from our multimodal networks, which are inherently undirected because the words are elicited in response to a shared stimulus. Nevertheless, we expect that the frequency of co‐occurrence is likely to be informative for future research focused on a finer analysis of the structure of those networks.

Third, our datasets, while diverse, are still thematically bounded. Even with modern tools like online crowdsourcing platforms and our semantic mining pipeline, collecting tags for the visual equivalent of a dataset like WordNet is still quite challenging, though not impossible with more general big data methodologies. Future work could investigate the persistence of the observed semantic regularities as the size of the datasets is scaled up. A potentially deeper criticism along these lines could argue that the communicative nature of STEP‐Tag, which encourages participants to succinctly describe the gist of observed stimuli, will always yield a subset of the true (possibly hierarchical) organization of semantic representations by excluding very general though uninformative superordinate categories like *object*. However, this argument is inconsistent with the available empirical data since the observed regularities persisted (i) in our free captioning data, where there is no explicit incentive to be succinct (in fact, the word *object* appears in all of our caption datasets with the exception of Emotional Prosody which makes sense), and (ii) in lexical networks, where there are no labeled stimuli. This highlights the importance of our findings in extending the literature by considering communicative, constrained, free, multimodal, and lexical elicitation settings.

Fourth, more work is needed to evaluate how semantic networks are reflected in neural data (Borge‐Holthoefer and Arenas, 2010). Our work sets the ground for such a future work by enriching the BOLD5000 dataset, which has publicly available neural data, with rich semantic labels that can be used to tap into this question.

Finally, our participant cohort was restricted to the United States and our annotations were limited to the English language and aggregated across participants. Equivalent cross‐cultural and individual‐differences research is necessary to determine the extent to which our findings generalize to non‐Western cohorts (Blasi, Henrich, Adamou, Kemmerer, & Majid, 2022) and vary across individuals (Marti, Wu, Piantadosi, & Kidd, 2023). This is particularly relevant for stimuli like those in the WikiArt dataset or music, where the semantics have been shown to vary cross‐culturally (Margulis, Wong, Turnbull, Kubit, & McAuley, 2022). We hope to explore these directions in future work.

A complete theory of human semantic organization must ultimately grapple with the complexities of real‐world domains. This, in turn, necessitates high‐quality and large‐scale psychological data. Our behavioral framework provides a new step toward achieving this goal. Finding that large‐scale regularities observed in free association networks and large lexical datasets are also reflected in the spontaneous descriptive taxonomies that arise in response to naturalistic stimuli sheds important new light on the processes underlying the structure of human semantic networks by directly linking it to the environment.

## Competing interests

The authors declare no competing interests.

## Ethics statement

All participants provided informed consent prior to participation in accordance with a Princeton University Institutional Review Board protocol (10859) and a Max Planck Society Ethics Council protocol (2021_42).

## Supporting information

Data S1

## Notes

1 We use the term *multimodal* to differentiate our networks from prior *lexical* semantic networks that are constructed purely based on textual data as in free associations.

2
https://wordnet.princeton.edu/

3
https://www.nltk.org/

4
https://pypi.org/project/pyspellchecker/

5
https://networkx.org/

6
https://github.com/fluhus/wordnet‐to‐json

7 https://osf.io/n63s2/overview/

8
https://pypi.org/project/wordfreq/
