# Supplementary material for: Characterizing the Large‐Scale Structure of Multimodal Semantic Networks
Source: Cogn Sci. 2025 Oct 23;49(10):e70131. doi: 10.1111/cogs.70131 (PMC12550224; doi:10.1111/cogs.70131)
Supplement: Supplementary file 1 — Data S1 [file COGS-49-e70131-s001.pdf]

Appendix A

Additional Details Regarding Stimulus Datasets

Bold5000 Indoor-Outdoor Scene Categories

ATM, HorseRaceTrack, ISS, MRI, RVinside, ShowJumping, airplanecabin, airportTerminal, alley, amusement, apartmentBuilding, appleorchard, applestore, aquarium, arcade, artStudio, attic, auto_showroom, backstage, backyard, baggageClaim, bakery, bakerykitchen, balconyIN, balconyOUT, ballroom, bambooforest, banquetHall, bar, barbershop, baseball, basement, basketballcourt, bathroomSink, baywindow, beach, bedroom, bikerack, bistrooutdoor, bleachers, boardwalk, botanicalGarden, bowlingalley, breakroom, brewery, bridalboutique, bridge, bus_shelter, cabana, cabin, cabinIndoors, cafeteria, campsite, campusquad, canyon, carFactory, cargo, carmechanic, carwash, casino, castle, cave, cemetery, checkoutcounter, chemicalplant, chickenCoop, childsroom, church, churchInside, circus, citystreet, classroom, cliff, closet, clothingstore, cockpit, coffeeshop, concert, concerthall, conference, constructionSite, copyroom, coralreef, cornfield, courthouse, courtroom, cubicle, dancestudio, deck, dentist, desertvegetation, diner, dining_car, diningroom, dinosaur, dock, doctor, dollhouse, dormroom, driveway, drycleaners, drymountains, dugout, elevatorOutside, exerciseequipment, eyeglassstore, fabricstore, factory, farm, farmersmarket, fireEscape, fireplace, firestation, fishmarket, fittingroom, flowershop, foodcourt, footballstadium, forestpath, fountain, frontDoor, frontFoyer, frontLawn, garbagedump, gasstation, gazebo, glaciers_outdoor, golfcourse, greenhouse, greenmountains, grocerystore, hallway, harbor, hardwarestore, hayfield, hedgemaze, highway, homegarage, homegarden, homeoffice, hometheater, horsebarn, hospitalroom, hotelRoom, hotellobby, hottub, hut, ice_rink, icecream, igloo, island, jail, jewelrystore, kindergarten_classroom, kitchen, lab, labClass, lake, laundromat, library_aisle, lightingstore, liquorstore, livingroom, lockerroom, lookout, mallindoor, martialartsclass, metroInside, minigolf, movie_theater, museum, musicStore, nailsalon, newsstand, nursery, observatory, office, olympicswimmingpool, onboat, operatingroom, pantry, park, parkinggarage, parkinglot, pasture, pavilion, pharmacy, photographer_studio, pier, pizzeria, planetarium, playground, playroom, pond, poolhall, porch, postoffice, promenadeDeck, pub, publicrestroom, racecarcourse, rainforest, readingroom, recordingstudio, restaurant, restaurantkitchen, rooftop, runway, ruralroad, sandDunes_desert_outdoor, sandbox, savanna, sculpturegarden, shoestore, shootingrange, shower, sidewalk, skatepark, skislope, snowmountains, soccerfield, sparoom, squashcourt, staircase, stream_outdoor, stripmall, sushibar, swimmingpool, tailor, tenniscourt, theater, ticket_counter, tollbooth, track, trainStation, treehouse, tvstudio, van_interior, volleyballcourt, waitingroom, warehouse, warehouseoutside, waterfall_outdoor, weightroom, whitewaterrapids, windmill, wineVineyard, winebarrel, wrestlingring, yogastudio.

Mini-Kinetics Video Categories

abseiling, air drumming, archery, arm wrestling, baking cookies, balloon blowing, barbequing, beatboxing, belly dancing, bench pressing, biking through snow, blowing glass, blowing out candles, bookbinding, bowling, braiding hair, breakdancing, brushing hair, brushing teeth, bungee jumping, busking, canoeing or kayaking, capoeira, catching or throwing baseball, catching or throwing frisbee, catching or throwing softball, cheerleading, chopping wood, clean and jerk, cleaning floor, climbing tree, contact juggling, cooking chicken, country line dancing, crawling baby, crossing river, crying, curling hair, cutting watermelon, dancing ballet, dancing gangnam style, dancing macarena, deadlifting, diving cliff, doing nails, dribbling basketball, driving car, driving tractor, dunking basketball, dying hair, eating burger, eating ice cream, eating spaghetti, feeding birds, feeding fish, feeding goats, filling eyebrows, finger snapping, flying kite, folding napkins, folding paper, front raises, giving or receiving award, golf driving, golf putting, gymnastics tumbling, hammer throw, headbanging, high jump, high kick, hitting baseball, hula hooping, hurling (sport), ice climbing, ice skating, javelin throw, jetskiing, juggling balls, jumping into pool, kicking field goal, kitesurfing, laughing, long jump, lunge, making pizza, making snowman, marching, massaging back, milking cow, motorcycling, mowing lawn, opening present, paragliding, parasailing, passing American football (in game), passing American football (not in game), petting animal (not cat), picking fruit, playing accordion, playing badminton, playing bagpipes, playing basketball, playing bass guitar, playing cello, playing chess, playing clarinet, playing cricket, playing didgeridoo, playing drums, playing guitar, playing harmonica, playing harp, playing ice hockey, playing paintball, playing poker, playing recorder, playing saxophone, playing squash or racquetball, playing tennis, playing trombone, playing trumpet, playing ukulele, playing violin, playing volleyball, playing xylophone, pole vault, presenting weather forecast, pull ups, pumping fist, punching bag, pushing car, pushing cart, reading book, riding elephant, riding or walking with horse, riding unicycle, robot dancing, rock climbing, roller skating, sailing, salsa dancing, scrambling eggs, scuba diving, shaking head, sharpening pencil, shaving head, shearing sheep, shot put, shoveling snow, shuffling cards, side kick, singing, situp, skateboarding, ski jumping, skiing (not slalom or crosscountry), slacklining, sled dog racing, smoking, smoking hookah, snatch weight lifting, snorkeling, snowboarding, snowkiting, somersaulting, spinning poi, spray painting, squat, sticking tongue out, stretching leg, surfing crowd, surfing water, swimming backstroke, swimming breast stroke, tai chi, tango dancing, tap dancing, tapping guitar, throwing axe, throwing discus, tobogganing, trapezing, trimming or shaving beard, triple jump, tying knot (not on a tie), unboxing, using computer, walking the dog, washing dishes, washing feet, washing hands, water skiing, waxing chest, waxing legs, weaving basket, welding, windsurfing, wrapping present, yoga, zumba.

Appendix B

Behavioral Prompts

Step-Tag

BOLD5K. 'In this game you will: Watch an image of an object or a scene and focus on its content. Rate tags that other players have given. Add new tags that you think are missing.'

Mini-Kinetics. 'In this game you will: Watch a video and focus on the activities happening. Rate tags that other players have given. Add new tags that you think are missing.'

WikiArt. 'In this game you will: Be presented with an abstract painting and you have to focus on the emotional content. Rate tags that other players have given. Add new tags that you think are missing.'

Emotional Prosody. 'In this game you will: Listen to a speech fragment and focus on the emotional content of the recording. Rate tags that other players have given. Add new tags that you think are missing.'

Free Captions

BOLD5K. 'In this experiment we are studying how people describe images. You will be presented with different images of objects and scenes and your task will be to describe their content.'

Mini-Kinetics. 'In this experiment we are studying how people describe activities in videos. You will be presented with different videos of activities and your task will be to describe their content.'

WikiArt. 'In this experiment we are studying how people describe abstract works of art. You will be presented with different abstract paintings and your task will be to describe their emotional content.'

Emotional Prosody. 'In this experiment we are studying how people describe emotions. You will be presented with different recordings of speakers and your task will be

to describe their emotions.'

Similarity Judgments

BOLD5K. 'In this experiment we are studying how people perceive images. In each round you will be presented with two different images of objects and/or scenes and your task will be to simply judge how similar they are.'

Mini-Kinetics. 'In this experiment we are studying how people perceive activities. In each round you will be presented with two different videos and your task will be to simply judge how similar are the activities in them.'

WikiArt. 'In this experiment we are studying how people perceive works of art. In each round you will be presented with two different abstract paintings and your task will be to simply judge how similar are the emotions they evoke.'

Emotional Prosody. 'In this experiment we are studying how people perceive emotions. In each round you will be presented with two different recordings and your task will be to simply judge how similar are the emotions of the speakers.'

Appendix C

Supplementary Information - STEP-Tag

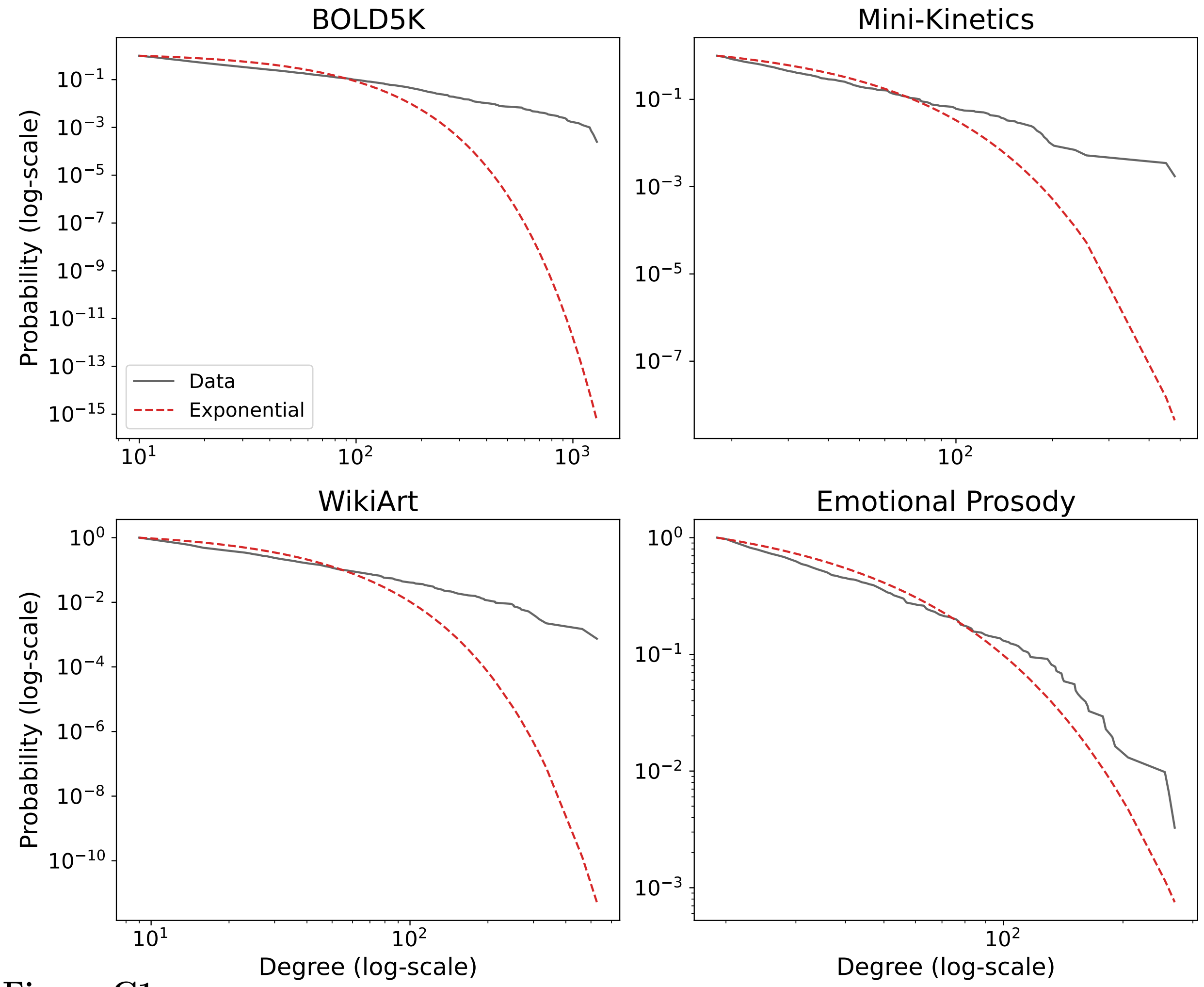

Figure C1

Complementary cumulative distribution function of the degree distribution for all STEP-Tag datasets on a log-log scale, along with an exponential model fit.

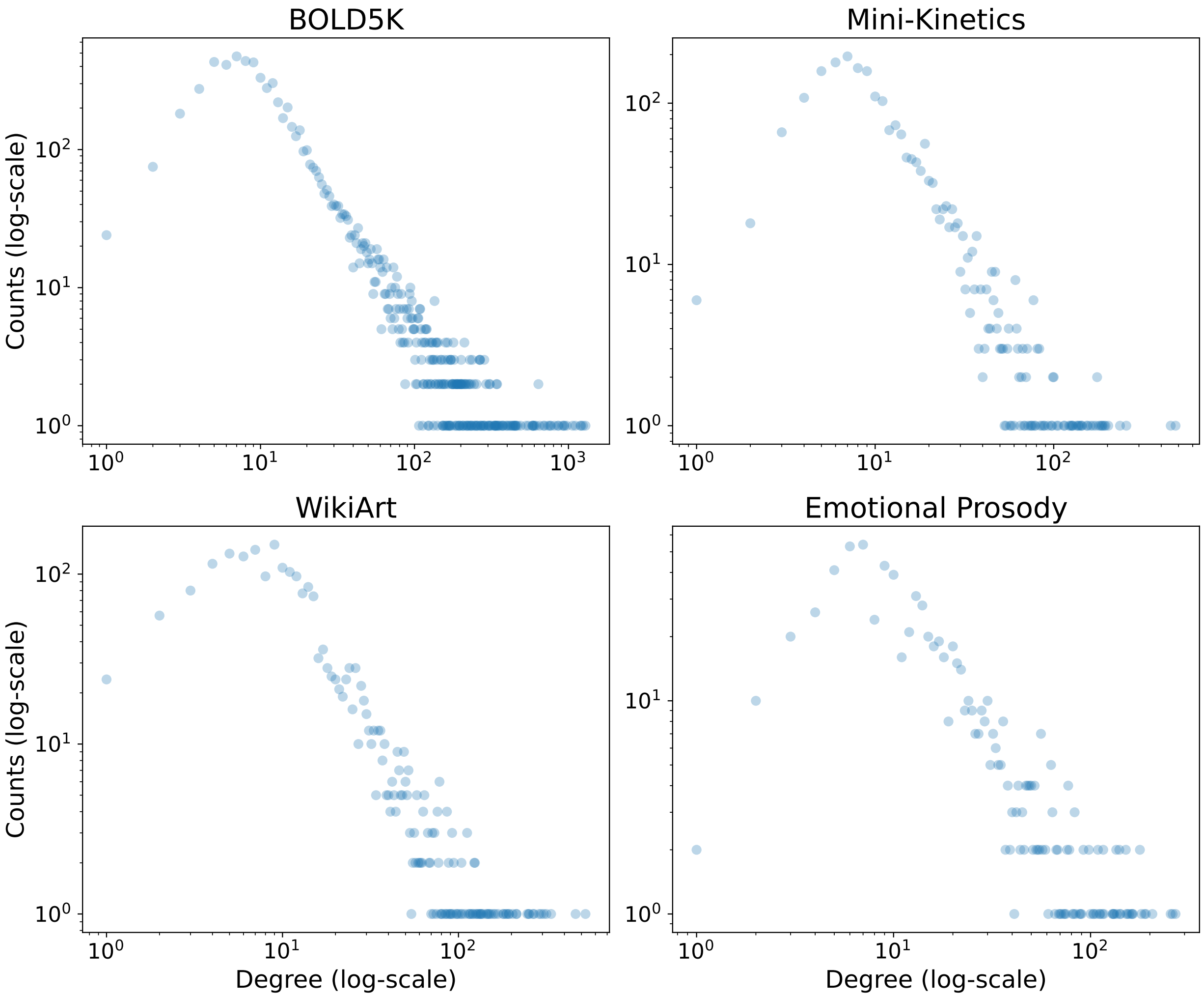

Figure C2

Node degree frequency on a log-log scale for the various STEP-Tag datasets.

Appendix D

Supplementary Information - Free Captions

Table D1

Graph statistics for the free captions multimodal semantic networks and their 95% confidence intervals.

Dataset	$\bar{d}$	$CI_{\bar{d}}$	$\bar{s}$	$CI_{\bar{s}}$	L	CI_L
BOLD5K	78.5	[77.6,79.6]	1.27%	[1.25,1.29]%	2.26	[2.25,2.27]
Mini-Kinetics	96.2	[94.4,98.3]	2.85%	[2.78,2.93]%	2.01	[2.01,2.02]
WikiArt	179.5	[177.1,181.9]	3.35%	[3.27,3.40]%	1.97	[1.97,1.97]
Prosody	98.9	[97.1,100.8]	4.15%	[4.05,4.31]%	1.96	[1.96,1.96]

Dataset	C	CI_C	σ	CI_σ	r_d	CI_{r_d}
BOLD5K	.764	[.760,.767]	62.7	[61.4,64.0]	-0.233	[-0.235,-0.231]
Mini-Kinetics	.783	[.779,.786]	27.7	[26.9,28.5]	-0.235	[-0.240, -0.231]
WikiArt	.786	[.783,.789]	23.5	[23.1,24.1]	-0.259	[-0.261, -0.257]
Prosody	.816	[.813,.818]	19.8	[19.1,20.4]	-0.311	[-0.316,-0.307]

Note: The measures are: average node degree $\bar{d}$, average sparsity $\bar{s} = \bar{d}/|G|$ where $|G|$ is the size of the network, L is the average shortest path length, C is the average clustering coefficient, σ is the small-worldness coefficient, and r_d is the degree assortativity. CI indicates 95% confidence intervals. See Methods for full details.

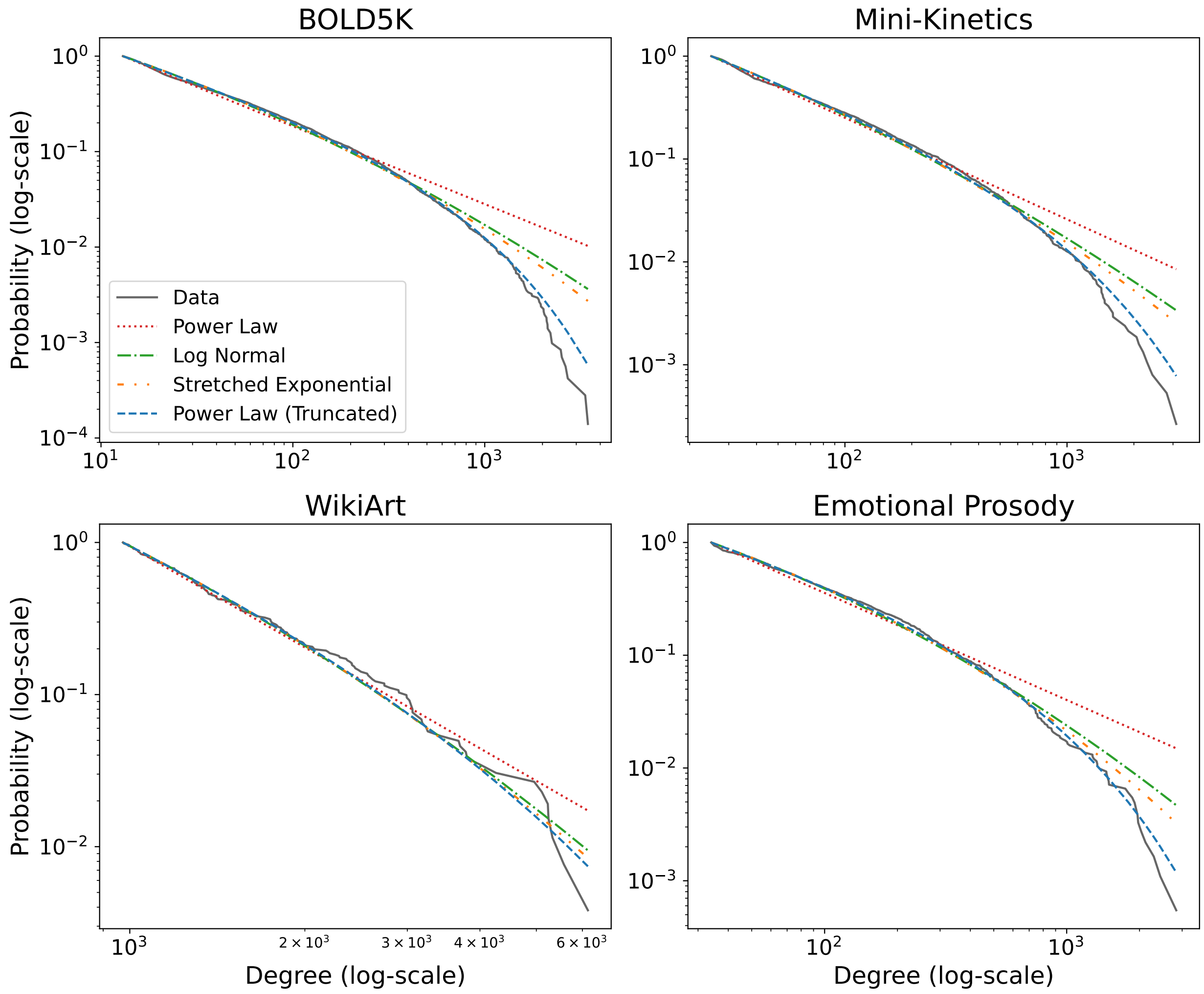

Figure D1

Complementary cumulative distribution function of the tail degree distribution (see Methods) for all free caption datasets on a log-log scale, along with different fitted models (exponential model provided separately in Supplementary Figure D2 due to poor fit).

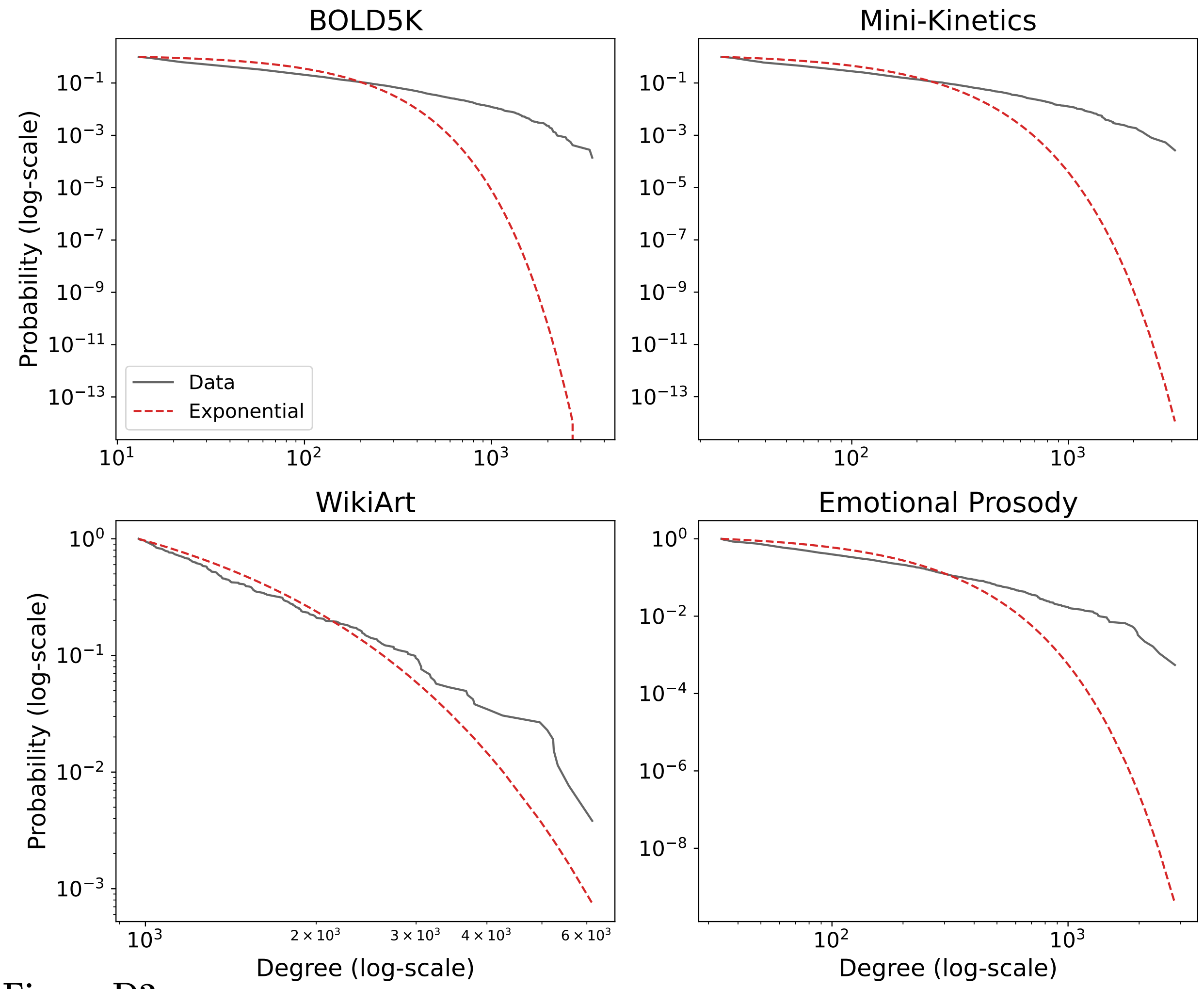

Figure D2

Complementary cumulative distribution function of the degree distribution for all caption datasets on a log-log scale, along with an exponential model fit.

Table D2

Top semantic hubs for the Free Caption Networks.

Dataset	Top Hub Words	Top Hub Degrees
BOLD5K	Look, White, Large, Two, Sit, Black, Red, Brown, Stand, Like	3450, 3340, 2709, 2639, 2522, 2489, 2240, 2219, 2199, 2127
Mini-Kinetics	Man, Person, Play, Young, People, Someone, Woman, Two, Show, Video	3103, 2809, 2423, 2290, 2201, 2104, 2056, 1889, 1822, 1710
WikiArt	Feel, Like, Look, Color, Paint, Make, Image, Picture, Art, Feeling	6133, 5577, 5331, 5247, 5233, 5110, 4957, 4263, 4019, 3803
Prosody	Sound, Speaker, Door, Man, Voice, Seem, Dog, Like, Woman, Kid	2836, 2436, 2290, 2117, 2032, 1974, 1962, 1933, 1908, 1865

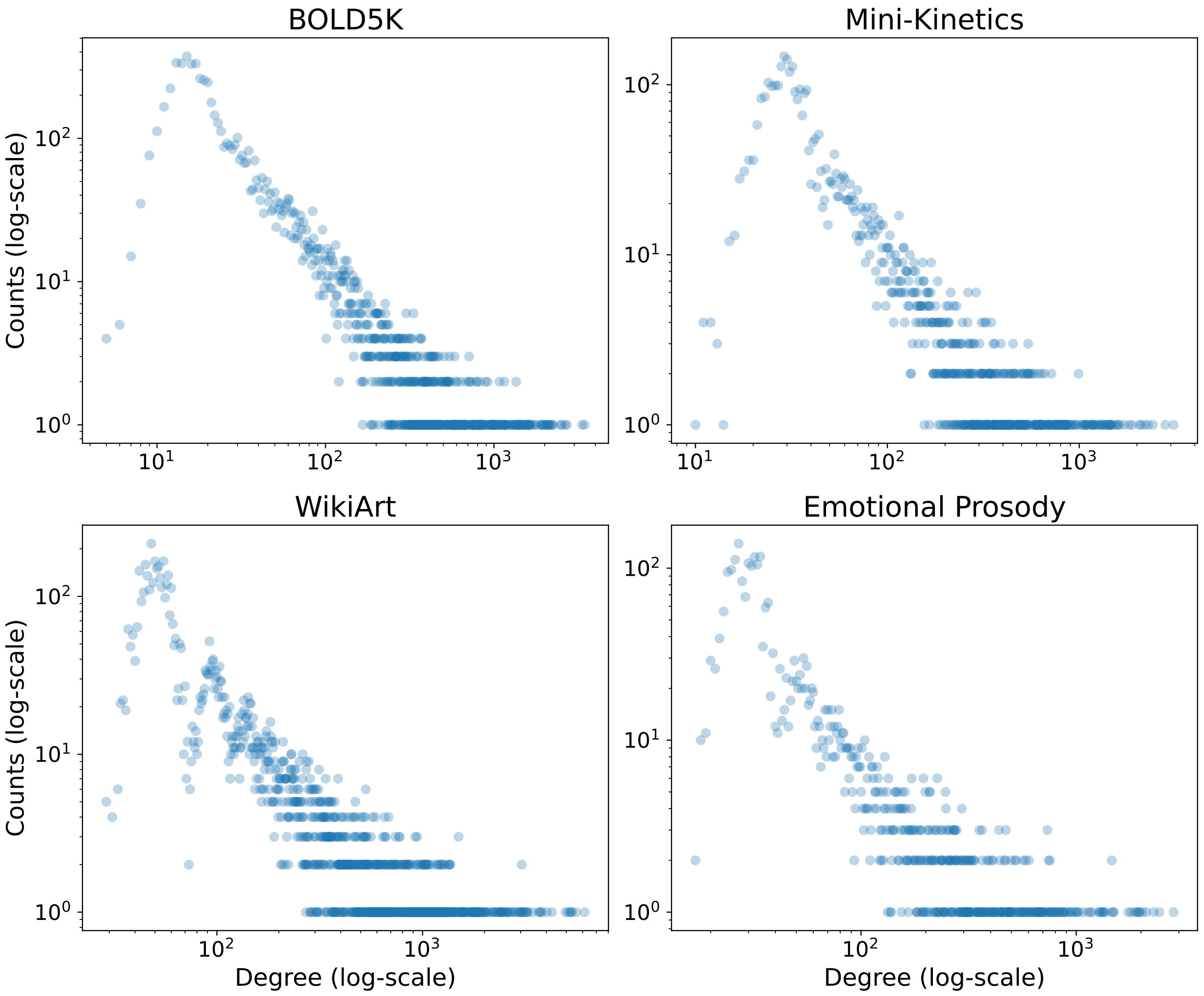

Figure D3

Node degree frequency on a log-log scale for the various free-text caption datasets.

Appendix E

Additional Network Statistics

Table E1

Number and fraction of nodes in largest connected component during bootstrapping over stimuli.

Dataset	N_{max}	$\mathrm{CI}_{N_{max}}$	$\bar{\Delta}_{max}$	$\mathrm{CI}_{\bar{\Delta}_{max}}$
BOLD5K (Tags)	5203	[5129, 5261]	.771	[.760, .779]
Mini-Kinetics (Tags)	1703	[1655, 1745]	.781	[.759, .800]
WikiArt (Tags)	1584	[1516, 1634]	.746	[.714, .769]
Prosody (Tags)	658	[629, 679]	.836	[.799, .863]
BOLD5K (Captions)	6203	[6129, 6284]	.799	[.789, .809]
Mini-Kinetics (Captions)	3374	[3302, 3444]	.792	[.775, .808]
WikiArt (Captions)	5365	[5258, 5454]	.797	[.781, .810]
Prosody (Captions)	2381	[2324, 2440]	.787	[.767, .806]

Note: The measures are: average number and fraction of nodes in the largest connected component N_{max} and $\bar{\Delta}_{max}$, respectively. CI indicates 95% confidence intervals. See Methods for full details. Note that the bootstrapped numbers are somewhat smaller than those computed when considering the full set of stimuli without bootstrapping. This is expected as sampling stimuli with replacement always results in some duplication, and since we consider unweighted graphs, a repeated stimulus does not result in new nodes, edges, or weights.

Appendix F

Clustering Controls

As an additional sanity check, we ran another popular (though slower in our case) clustering algorithm: the Clauset-Newman-Moore (CNM) algorithm (Clauset et al., 2004). This was done using the `greedy_modularity_communities` method in the `networkx` package. We found that CNM generally underperformed Louvain as indicated by lower modularity scores relative to Louvain ($.33 - .42$ for STEP-Tag, and $.10 - .16$ for Captions), and the detection of many small clusters that contain ≤ 5 nodes (Tables F3-F4). Nonetheless, the Louvain and CNM clustering solutions did exhibit some degree of correlation in structure as indicated by positive adjusted Rand indices ($.20 - .61$ for STEP-Tag, and $.15 - .34$ for Captions; recall that an ARI of 0 corresponds to random clustering). See Supplementary Table F1-F4 for additional details.

Table F1

Louvain leading clusters (STEP-Tag), their top words and their within-cluster degree.

Dataset	Modularity	# Clusters	Cluster Size	Top Words	In-cluster Degree
Bold5K	0.386	14	1308	[table, window, chair, lights]	[394, 371, 338, 326]
Bold5K	0.386	14	1238	[grass, man, sky, green]	[401, 364, 338, 325]
Bold5K	0.386	14	1096	[white, trees, brown, dog]	[295, 294, 274, 268]
Mini-Kinetics	0.511	13	344	[music, performance, dance, stage]	[223, 113, 67, 65]
Mini-Kinetics	0.511	13	297	[baby, child, girl, kid]	[90, 85, 81, 52]
Mini-Kinetics	0.511	13	258	[sports, game, competition, field]	[105, 99, 92, 66]
WikiArt	0.399	15	372	[red, colorful, white, yellow]	[114, 103, 97, 87]
WikiArt	0.399	15	369	[calm, water, serene, peaceful]	[154, 123, 118, 116]
WikiArt	0.399	15	355	[abstract, black, messy, confusing]	[194, 90, 63, 61]
Prosody	0.438	4	238	[calm, relaxed, bored, neutral]	[175, 120, 120, 118]
Prosody	0.438	4	198	[happy, excited, surprised, amused]	[103, 91, 84, 65]
Prosody	0.438	4	184	[annoyed, angry, mad, furious]	[138, 124, 93, 93]

Table F2
Louvain leading clusters (Captions), their top words and their within-cluster degree.

Dataset	Modularity	# Clusters	Cluster Size	Top Words	In-cluster Degree
Bold5K	0.207	10	1737	[look, white, brown, black]	[1020, 912, 843, 754]
Bold5K	0.207	10	1528	[large, blue, front, water]	[694, 550, 521, 500]
Bold5K	0.207	10	1503	[room, wall, color, show]	[685, 558, 541, 515]
Mini-Kinetics	0.208	7	853	[play, music, people, perform]	[610, 572, 571, 499]
Mini-Kinetics	0.208	7	732	[person, someone, water, rid]	[599, 510, 395, 378]
Mini-Kinetics	0.208	7	724	[man, video, show, use]	[549, 396, 395, 333]
WikiArt	0.124	8	1849	[like, color, paint, look]	[1626, 1612, 1562, 1534]
WikiArt	0.124	8	1691	[feel, image, woman, man]	[1533, 1239, 1065, 919]
WikiArt	0.124	8	1616	[shape, white, feeling, line]	[1137, 1123, 996, 964]
Prosody	0.168	5	1018	[speaker, calm, voice, bore]	[851, 794, 777, 665]
Prosody	0.168	5	832	[sound, angry, door, man]	[768, 647, 606, 578]
Prosody	0.168	5	578	[seem, surprise, dog, like]	[410, 410, 405, 387]

Table F3

CNM leading clusters (STEP-Tag), their top words and their within-cluster degree.

Dataset	Mod.	# Clusters	Cluster Size	Top Words	In-cluster Degree
Bold5K	0.333	70	2700	[trees, grass, sky, green]	[942, 907, 869, 727]
Bold5K	0.333	70	2360	[table, lights, food, chair]	[658, 462, 453, 446]
Bold5K	0.333	70	1120	[man, woman, white, baseball]	[320, 260, 184, 126]
Mini-Kinetics	0.423	29	597	[baby, kid, child, snow]	[124, 122, 113, 106]
Mini-Kinetics	0.423	29	567	[music, man, woman, performance]	[194, 134, 99, 90]
Mini-Kinetics	0.423	29	514	[gym, competition, sports, game]	[158, 143, 121, 100]
WikiArt	0.364	31	770	[abstract, red, colorful, black]	[287, 216, 193, 186]
WikiArt	0.364	31	566	[calm, water, peaceful, serene]	[201, 150, 136, 134]
WikiArt	0.364	31	326	[dark, painting, sadness, scared]	[126, 103, 66, 53]
Prosody	0.396	5	300	[upset, annoyed, angry, loud]	[171, 170, 151, 119]
Prosody	0.396	5	284	[calm, relaxed, neutral, bored]	[194, 133, 132, 131]
Prosody	0.396	5	196	[happy, excited, surprised, confused]	[94, 88, 87, 63]

Table F4
CNM leading clusters (Captions), their top words and their within-cluster degree.

Dataset	Mod.	# Clusters	Cluster Size	Top Words	In-cluster Degree
Bold5K	0.163	28	3439	[black, stand, brown, man]	[1328, 1311, 1252, 1152]
Bold5K	0.163	28	3135	[large, table, sit, show]	[1203, 1195, 1155, 1074]
Bold5K	0.163	28	925	[white, look, two, woman]	[348, 331, 251, 239]
Mini-Kinetics	0.177	17	1728	[play, people, perform, two]	[1302, 1204, 1000, 959]
Mini-Kinetics	0.177	17	1698	[person, young, someone, woman]	[1153, 981, 961, 960]
Mini-Kinetics	0.177	17	679	[man, water, rid, go]	[555, 327, 278, 264]
WikiArt	0.099	17	2830	[feel, paint, look, like]	[2599, 2208, 2201, 2200]
WikiArt	0.099	17	2692	[shape, different, line, yellow]	[2011, 1709, 1640, 1512]
WikiArt	0.099	17	1080	[color, art, black, white]	[884, 614, 574, 570]
Prosody	0.143	11	1229	[upset, angry, surprise, scar]	[849, 745, 694, 620]
Prosody	0.143	11	1215	[speaker, door, calm, voice]	[1000, 927, 912, 884]
Prosody	0.143	11	543	[sound, like, kid, talk]	[510, 325, 324, 279]

Appendix G

Computing Text and Stimulus Embeddings

To construct a similarity prediction from a pair of stimuli x_i, x_j or their text descriptors t_i, t_j we embedded them into vector representations v_i, v_j using a suitable embedding model, and then computed their cosine similarity to derive a prediction $\frac{v_i \cdot v_j}{||v_i|| ||v_j||}$ where $|| \cdot ||$ is the Euclidean norm. We considered the same set of models as Marjieh et al. (2023). Our stimuli cover three perceptual modalities, namely, images, audio, and video, in addition to text, each of which requiring different models. We describe these below.

Stimulus Embedding Models

Images. The image models comprised 569 pre-trained models from the `pytorch-image-models` package `timm` (Wightman, 2019). This package contains an extensive and diverse set of pre-trained models that span different architectures, model sizes, and popular training sets (e.g., ImageNet21K). The models were published between 2014-2022 and cover different training objectives (e.g., fine-tuning, self-supervision, and weak supervision) as well as common neural architectures (e.g., VGG, ResNet, Inception, and Transformer). Extensive details of all models can be accessed via this link[1]. For all models, the last layer was used as the stimulus embedding. The best performing model for each image dataset were: Bold5K: Vision Transformer (Dosovitskiy et al., 2020; Version `vit_large_patch16_384`[2]), WikiArt: ConvNeXt (Liu et al., 2022; Version `convnext_large_in22ft1k`[3]).

Audio. The audio models comprised 36 pre-trained models available through the `torchaudio` package (Yang et al., 2022). Specifically, the set included all wav2vec 2.0 (Baevski et al., 2020) and HuBERT (Hsu et al., 2021) models available. These included variants that were specialized on either emotion recognition or speaker identification. The

[1] https://github.com/huggingface/pytorch-image-models

[2] https://huggingface.co/timm/vit_large_patch16_384.augreg_in21k_ft_in1k

[3] https://huggingface.co/timm/convnext_large.fb_in22k_ft_in1k_384

set also included WavLM (Chen et al., 2022) and data2vec (Baevski et al., 2022) audio models. As with the images the last layers were used to generate embeddings. The best performing model for the Emotional Prosody dataset was wav2vec 2.0 (Baevski et al., 2020; Version `wav2vec2_large_lv60k`[4]).

Video. Embedding models for video comprised 6 pre-trained models from the `PyTorchVideo` package (Fan et al., 2021). These included three variants of X3d (an architecture that initially processes the video as 2D images and then expands across different axes; Feichtenhofer, 2020), Slow (a 3D ResNet; Feichtenhofer et al., 2019) and two variants of SlowFast (a two-path model for slow and fast feature processing; Feichtenhofer et al., 2019). Last layers were used as with audio and images. The best performing model for the Mini-Kinetics dataset was SlowFast (Feichtenhofer et al., 2019; Version `slowfast_r50`[5]).

Text Embedding Models

Tags. To embed tags, we used ConceptNet NumberBatch, a word embedding model that is trained on the ConceptNet knowledge graph while leveraging other common word embedding models such as word2vec and GloVe (Speer et al., 2017). Given a set of tag descriptors for a given stimulus, a single embedding vector was generated by averaging the tags.

Captions. Four text embedding models were considered for embedding captions. These were accessed through HuggingFace: `bert-base-uncased`[6], `deberta-xlarge-mnli`[7], `sup-simcse-bert-base-uncased`[8], and `sup-simcse-roberta-large`[9]. As with the stimulus embedding models, the last layers were used to derive embeddings. Moreover, we

[4] https://pytorch.org/audio/stable/generated/torchaudio.models.Wav2Vec2Model.html

[5] https://pytorch.org/hub/facebookresearch_pytorchvideo_slowfast/

[6] https://huggingface.co/google-bert/bert-base-uncased

[7] https://huggingface.co/microsoft/deberta-xlarge-mnli

[8] https://huggingface.co/princeton-nlp/sup-simcse-bert-base-uncased

[9] https://huggingface.co/princeton-nlp/sup-simcse-roberta-large

averaged the embeddings of multiple available captions per stimulus into a single embedding. The best performing models were SimCSE RoBERTa (Version `sup-simcse-roberta-large`; Gao et al., 2021) for BOLD5K, Mini-Kinetics and Emotional Prosody, and BERT (Version `bert-base-uncased`; Devlin et al., 2018) for WikiArt.

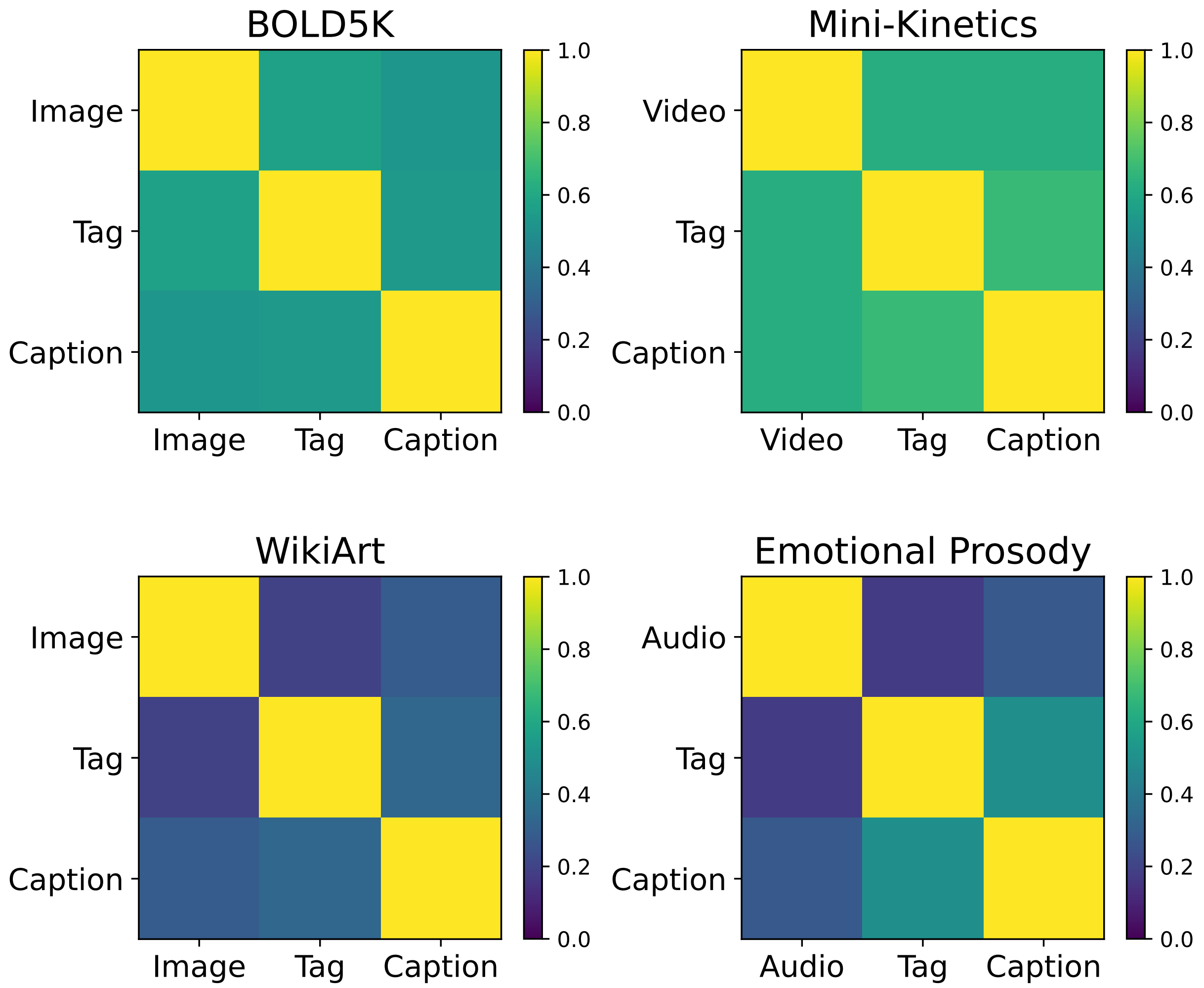

Figure G1
Pearson correlations between different similarity predictors across domains.

Appendix H

Additional Power-Law Analysis Metrics

Table H1

Full list of Log-likelihood ratio tests for the STEP-Tag degree distributions.

Dataset	Base	Alternative	LLR	p	Conclusion
BOLD5K	Power Law	Exponential	1368.836	0.0000	Power Law
BOLD5K	Power Law	Log-Normal	-26.752	0.0000	Log-Normal
BOLD5K	Power Law	Stretched Exp.	-29.228	0.0000	Stretched Exp.
BOLD5K	Power Law	Power Law (Tr.)	-39.191	0.0000	Power Law (Tr.)
BOLD5K	Power Law (Tr.)	Exponential	1408.027	0.0000	Power Law (Tr.)
BOLD5K	Power Law (Tr.)	Log-Normal	12.439	0.0000	Power Law (Tr.)
BOLD5K	Power Law (Tr.)	Stretched Exp.	9.962	0.0000	Power Law (Tr.)
BOLD5K	Power Law (Tr.)	Power Law	39.191	0.0000	Power Law (Tr.)
Mini-Kinetics	Power Law	Exponential	76.443	0.0001	Power Law
Mini-Kinetics	Power Law	Log-Normal	-2.229	0.1856	Inconclusive
Mini-Kinetics	Power Law	Stretched Exp.	-2.352	0.2378	Inconclusive
Mini-Kinetics	Power Law	Power Law (Tr.)	-3.028	0.0139	Power Law (Tr.)
Mini-Kinetics	Power Law (Tr.)	Exponential	79.470	0.0000	Power Law (Tr.)
Mini-Kinetics	Power Law (Tr.)	Log-Normal	0.799	0.1653	Inconclusive
Mini-Kinetics	Power Law (Tr.)	Stretched Exp.	0.676	0.2353	Inconclusive
Mini-Kinetics	Power Law (Tr.)	Power Law	3.028	0.0139	Power Law (Tr.)
WikiArt	Power Law	Exponential	268.948	0.0000	Power Law
WikiArt	Power Law	Log-Normal	-11.462	0.0026	Log-Normal
WikiArt	Power Law	Stretched Exp.	-12.049	0.0019	Stretched Exp.
WikiArt	Power Law	Power Law (Tr.)	-13.484	0.0000	Power Law (Tr.)
WikiArt	Power Law (Tr.)	Exponential	282.432	0.0000	Power Law (Tr.)
WikiArt	Power Law (Tr.)	Log-Normal	2.022	0.1412	Inconclusive
WikiArt	Power Law (Tr.)	Stretched Exp.	1.435	0.2174	Inconclusive
WikiArt	Power Law (Tr.)	Power Law	13.484	0.0000	Power Law (Tr.)
Prosody	Power Law	Exponential	0.748	0.9313	Inconclusive
Prosody	Power Law	Log-Normal	-11.987	0.0012	Log-Normal
Prosody	Power Law	Stretched Exp.	-13.272	0.0007	Stretched Exp.
Prosody	Power Law	Power Law (Tr.)	-14.320	0.0000	Power Law (Tr.)
Prosody	Power Law (Tr.)	Exponential	15.068	0.0034	Power Law (Tr.)
Prosody	Power Law (Tr.)	Log-Normal	2.333	0.0054	Power Law (Tr.)
Prosody	Power Law (Tr.)	Stretched Exp.	1.048	0.0269	Power Law (Tr.)
Prosody	Power Law (Tr.)	Power Law	14.320	0.0000	Power Law (Tr.)

Note: Tr. indicates truncation (see Methods). LLR is the log-likelihood ratio test. Suitable nested log-likelihood tests were used when one of the functions was a subset of the other (e.g., when comparing a truncated power law with a regular power law; Alstott et al., 2014). p indicates the p-value of the test. Significance was determined with a $p < 0.05$ threshold. A value of 0.0000 indicates $p < 0.0001$.

Table H2

Full list of Log-likelihood ratio tests for the Free Captions degree distributions.

Dataset	Base	Alternative	LLR	p	Conclusion
BOLD5K	Power Law	Exponential	3373.722	0.0000	Power Law
BOLD5K	Power Law	Log-Normal	-59.862	0.0000	Log-Normal
BOLD5K	Power Law	Stretched Exp.	-68.366	0.0000	Stretched Exp.
BOLD5K	Power Law	Power Law (Tr.)	-106.770	0.0000	Power Law (Tr.)
BOLD5K	Power Law (Tr.)	Exponential	3480.492	0.0000	Power Law (Tr.)
BOLD5K	Power Law (Tr.)	Log-Normal	46.908	0.0000	Power Law (Tr.)
BOLD5K	Power Law (Tr.)	Stretched Exp.	38.404	0.0000	Power Law (Tr.)
BOLD5K	Power Law (Tr.)	Power Law	106.770	0.0000	Power Law (Tr.)
Mini-Kinetics	Power Law	Exponential	1256.682	0.0000	Power Law
Mini-Kinetics	Power Law	Log-Normal	-21.788	0.0000	Log-Normal
Mini-Kinetics	Power Law	Stretched Exp.	-24.892	0.0000	Stretched Exp.
Mini-Kinetics	Power Law	Power Law (Tr.)	-41.586	0.0000	Power Law (Tr.)
Mini-Kinetics	Power Law (Tr.)	Exponential	1298.268	0.0000	Power Law (Tr.)
Mini-Kinetics	Power Law (Tr.)	Log-Normal	19.798	0.0000	Power Law (Tr.)
Mini-Kinetics	Power Law (Tr.)	Stretched Exp.	16.694	0.0000	Power Law (Tr.)
Mini-Kinetics	Power Law (Tr.)	Power Law	41.586	0.0000	Power Law (Tr.)
WikiArt	Power Law	Exponential	8.328	0.1305	Inconclusive
WikiArt	Power Law	Log-Normal	-1.517	0.2358	Inconclusive
WikiArt	Power Law	Stretched Exp.	-1.714	0.2201	Inconclusive
WikiArt	Power Law	Power Law (Tr.)	-2.186	0.0366	Power Law (Tr.)
WikiArt	Power Law (Tr.)	Exponential	10.514	0.0127	Power Law (Tr.)
WikiArt	Power Law (Tr.)	Log-Normal	0.669	0.0080	Power Law (Tr.)
WikiArt	Power Law (Tr.)	Stretched Exp.	0.472	0.0092	Power Law (Tr.)
WikiArt	Power Law (Tr.)	Power Law	2.186	0.0366	Power Law (Tr.)
Prosody	Power Law	Exponential	437.774	0.0000	Power Law
Prosody	Power Law	Log-Normal	-25.179	0.0000	Log-Normal
Prosody	Power Law	Stretched Exp.	-29.076	0.0000	Stretched Exp.
Prosody	Power Law	Power Law (Tr.)	-38.791	0.0000	Power Law (Tr.)
Prosody	Power Law (Tr.)	Exponential	476.565	0.0000	Power Law (Tr.)
Prosody	Power Law (Tr.)	Log-Normal	13.612	0.0000	Power Law (Tr.)
Prosody	Power Law (Tr.)	Stretched Exp.	9.715	0.0000	Power Law (Tr.)
Prosody	Power Law (Tr.)	Power Law	38.791	0.0000	Power Law (Tr.)

Note: Tr. indicates truncation (see Methods). LLR is the log-likelihood ratio test. Suitable nested log-likelihood tests were used when one of the functions was a subset of the other (e.g., when comparing a truncated power law with a regular power law; Alstott et al., 2014). p indicates the p-value of the test. Significance was determined with a $p < 0.05$ threshold. A value of 0.0000 indicates $p < 0.0001$.

Table H3

Additional power-law analysis metrics.

Dataset	x_{min}	$\mathrm{CI}_{x_{min}}$	$N_{\geq x_{min}}$	$\mathrm{CI}_{N_{\geq x_{min}}}$
BOLD5K (Tags)	9.9	[9.0, 12.0]	4134.0	[3481.4, 4508.0]
Mini-Kinetics (Tags)	18.6	[11.0, 25.0]	583.7	[354.9, 1059.3]
WikiArt (Tags)	15.5	[9.0, 42.0]	963.9	[220.0, 1374.0]
Prosody (Tags)	20.4	[11.0, 48.0]	345.6	[119.0, 490.0]
BOLD5K (Captions)	31.7	[12.0, 295.0]	6700.0	[531.0, 7367.0]
Mini-Kinetics (Captions)	27.4	[24.0, 26.0]	3744.8	[3650.0, 3891.0]
WikiArt (Captions)	524.1	[81.0, 1144.1]	1706.2	[195.0, 3415.0]
Prosody (Captions)	93.4	[22.0, 438.0]	1371.5	[149.0, 2938.0]

Note: The measures are: average detected minimum degree above which the scaling regime (tail) starts x_{min}, and the average number of nodes above x_{min} that are included in the scaling regime (see Methods; Alstott et al., 2014). CI indicates 95% confidence intervals. See Methods for full details.

Table H4

Maximum likelihood power law parameters (normal and truncated).

Dataset	α	CI_{α}	$\hat{\alpha}$	$\mathrm{CI}_{\hat{\alpha}}$	λ	CI_{λ}
BOLD5K (Tags)	2.000	[1.962, 2.037]	1.846	[1.791, 1.905]	0.001	[0.001, 0.002]
Mini-Kinetics (Tags)	2.580	[2.296, 2.799]	2.301	[1.920, 2.598]	0.004	[0.001, 0.009]
WikiArt (Tags)	2.324	[2.153, 2.720]	2.012	[1.000, 2.445]	0.004	[0.001, 0.018]
Prosody (Tags)	2.288	[1.996, 3.052]	1.490	[1.000, 1.971]	0.009	[0.005, 0.018]
BOLD5K (Captions)	1.864	[1.779, 2.605]	1.674	[1.603, 1.960]	0.001	[0.001, 0.001]
Mini-Kinetics (Captions)	1.989	[1.951, 2.019]	1.808	[1.747, 1.859]	0.001	[0.000, 0.001]
WikiArt (Captions)	2.623	[1.967, 3.496]	2.116	[1.573, 3.041]	0.000	[0.000, 0.001]
Prosody (Captions)	2.128	[1.907, 3.046]	1.728	[1.000, 2.423]	0.001	[0.000, 0.002]

Note: The parameters are defined as follows: Normal: $x^{-\alpha}$, Truncated: $x^{-\hat{\alpha}}e^{-\lambda x}$. CI indicates 95% confidence intervals bootstrapped by sampling from the empirical degree distributions with replacement and repeating 1,000 times. See Methods for full details.

Table H5

Pure power-law tail plausibility test.

Dataset	D	p	Conclusion
BOLD5K (Tags)	0.020907	0.001	Fail
Mini-Kinetics (Tags)	0.024592	0.376	Pass
WikiArt (Tags)	0.034183	0.000	Fail
Prosody (Tags)	0.064702	0.000	Fail
BOLD5K (Captions)	0.038699	0.000	Fail
Mini-Kinetics (Captions)	0.032463	0.000	Fail
WikiArt (Captions)	0.037652	0.240	Pass
Prosody (Captions)	0.048563	0.000	Fail

Note: The entries are: best Kolmogorov-Smirnov (KS) statistic D between the fitted power law model and the empirical tail above x_{min} (see Methods), test p-value p, and the test conclusion (the pure power law is ruled out when $p \leq 0.1$; Clauset et al., 2009; see also Alstott et al., 2014). The plausibility test is conducted through a bootstrapping procedure whereby equal-sized data replicas are produced such that below x_{min} they have the same distribution as the empirical data (by sampling with replacement), and above x_{min} they are sampled from the fitted (perfect) power-law tail distribution. The power-law fitting procedure is then applied to each of those replicas, each resulting in its own KS statistic D_{rep}. The p-value is then computed by estimating the fraction of times the replica KS statistic D_{rep} exceeds (i.e., under-performs) the empirical value D. We generated 1,000 bootstrap replicas per condition. Consistent with our log-likelihood analysis, we see that a pure power law does not pass the test in most conditions.

Appendix I

Lexical Network Analysis

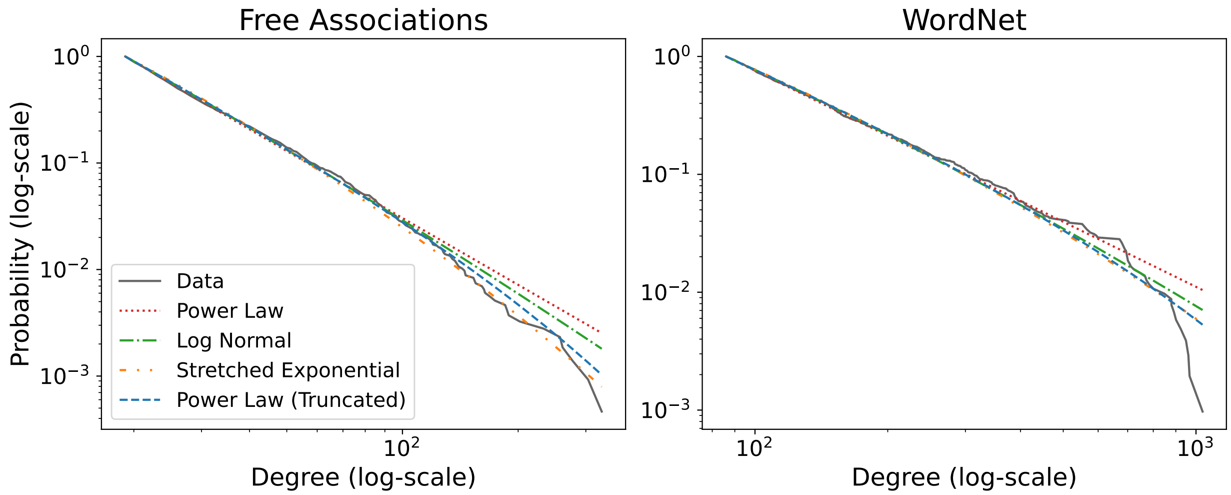

Figure I1

Complementary cumulative distribution function of the tail degree distribution (see Methods) for two lexical networks on a log-log scale, along with different fitted models (exponential model provided separately in Supplementary Figure I2 due to poor fit).

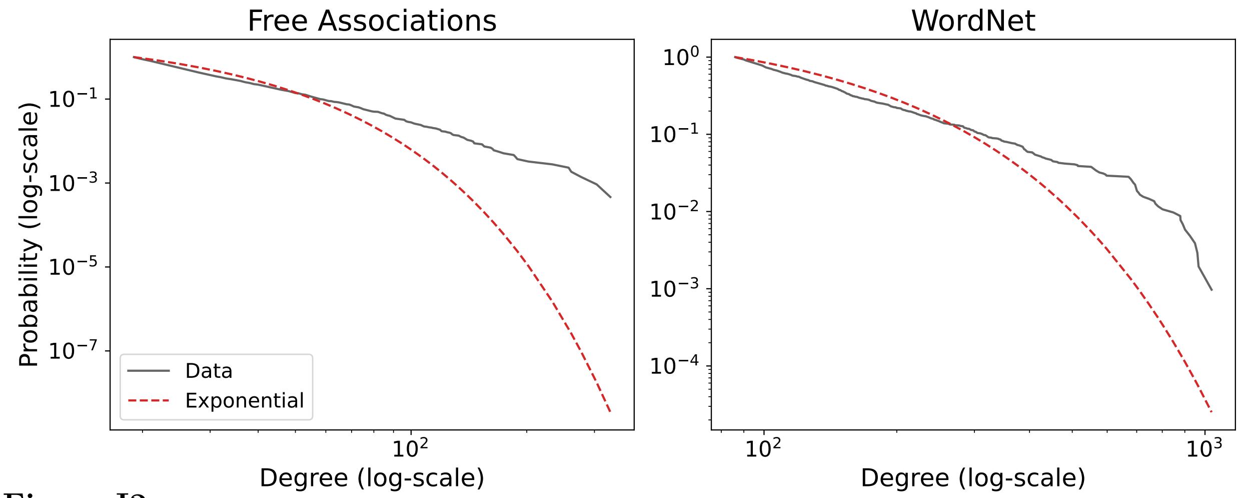

Figure I2

Complementary cumulative distribution function of the degree distribution for two lexical networks on a log-log scale, along with an exponential model fit.

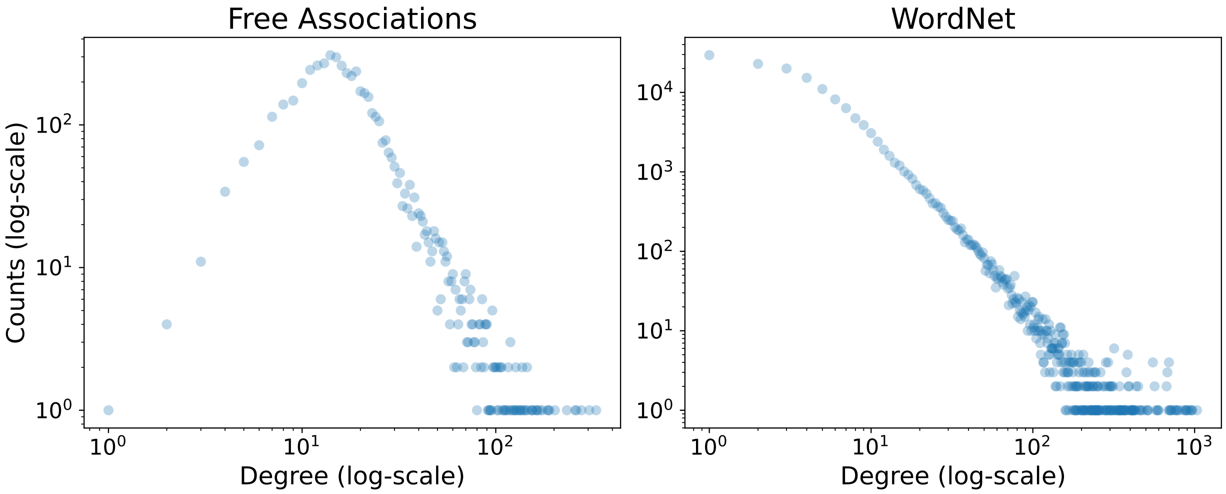

Figure I3

Node degree frequency on a log-log scale for two lexical networks.

Table I1

Full list of Log-likelihood ratio tests for the lexical degree distributions.

Dataset	Base	Alternative	LLR	p	Conclusion
Free Association	Power Law	Exponential	221.716	0.0000	Power Law
Free Association	Power Law	Log-Normal	-0.950	0.3672	Inconclusive
Free Association	Power Law	Stretched Exp.	1.683	0.6025	Inconclusive
Free Association	Power Law	Power Law (Tr.)	-2.769	0.0186	Power Law (Tr.)
Free Association	Power Law (Tr.)	Exponential	224.485	0.0000	Power Law (Tr.)
Free Association	Power Law (Tr.)	Log-Normal	1.819	0.0028	Power Law (Tr.)
Free Association	Power Law (Tr.)	Stretched Exp.	4.452	0.0118	Power Law (Tr.)
Free Association	Power Law (Tr.)	Power Law	2.769	0.0186	Power Law (Tr.)
WordNet	Power Law	Exponential	108.020	0.0000	Power Law
WordNet	Power Law	Log-Normal	-1.735	0.2037	Inconclusive
WordNet	Power Law	Stretched Exp.	-1.586	0.4607	Inconclusive
WordNet	Power Law	Power Law (Tr.)	-3.539	0.0078	Power Law (Tr.)
WordNet	Power Law (Tr.)	Exponential	111.559	0.0000	Power Law (Tr.)
WordNet	Power Law (Tr.)	Log-Normal	1.804	0.0000	Power Law (Tr.)
WordNet	Power Law (Tr.)	Stretched Exp.	1.953	0.0062	Power Law (Tr.)
WordNet	Power Law (Tr.)	Power Law	3.539	0.0078	Power Law (Tr.)

Note: Tr. indicates truncation (see Methods). LLR is the log-likelihood ratio test. Suitable nested log-likelihood tests were used when one of the functions was a subset of the other (e.g., when comparing a truncated power law with a regular power law; Alstott et al., 2014). p indicates the p-value of the test. Significance was determined with a $p < 0.05$ threshold. A value of 0.0000 indicates $p < 0.0001$.

Table I2

Additional power-law analysis metrics for the lexical networks.

Dataset	x_{min}	$\mathrm{CI}_{x_{min}}$	$N_{\geq x_{min}}$	$\mathrm{CI}_{N_{\geq x_{min}}}$
Free Association	18.5	[18.0, 21.0]	2273.8	[1789.8, 2510.2]
WordNet	22.9	[7.0, 66.0]	25874.6	[1633.9, 40008.0]

Note: The measures are: average detected minimum degree above which the scaling regime (tail) starts x_{min}, and the average number of nodes above x_{min} that are included in the scaling regime (see Methods; Alstott et al., 2014). CI indicates 95% confidence intervals bootstrapped by sampling from the empirical degree distributions with replacement and repeating 1,000 times. See Methods for full details.

Table I3

Maximum likelihood power law parameters (normal and truncated) for lexical networks.

Dataset	α	CI_α	$\hat{\alpha}$	$\mathrm{CI}_{\hat{\alpha}}$	λ	CI_λ
Free Association	3.057	[2.970, 3.153]	2.852	[2.679, 3.020]	0.004	[0.002, 0.006]
WordNet	2.461	[2.298, 2.841]	2.290	[1.000, 2.707]	0.004	[0.000, 0.047]

Note: The parameters are defined as follows: Normal: $x^{-\alpha}$, Truncated: $x^{-\hat{\alpha}}e^{-\lambda x}$. CI indicates 95% confidence intervals bootstrapped by sampling from the empirical degree distributions with replacement and repeating 1,000 times. See Methods for full details.

Appendix J

Semantic Overlap Between Lexical and Multimodal Networks

Shared Hubs with the Free Association Network

BOLD5K (Tags): white, green, man, water, people, red, tree, black, wood, food, light, dog, person, car, animal, dirt, house, child, clean, bird, girl, room.

Mini-Kinetics (Tags): man, music, girl, water, child, game, baby, people, fun, hair, ball, tree, play, food, cold.

WikiArt (Tags): red, black, dark, white, green, water, happy, sad, man, people, tree, old, love, person, boring.

Prosody (Tags): loud, sad, happy, man, girl.

BOLD5K (Captions): white, black, red, show, green, man, small, people, person, old, light, water, tree, good, room, play, nice, food, dog, long, big, wood, dark, house.

Mini-Kinetics (Captions): man, person, play, people, show, music, girl, child, talk, small, white, together, game, ball, water, black, room, high, red, work, run, old, hit, hair.

WikiArt (Captions): white, show, black, nice, dark, good, red, people, happy, life, man, light, work, green, sad, person, water, together, time, tree, small, love, old.

Prosody (Captions): man, dog, talk, person, happy, sad, girl, loud.

Shared Hubs with the WordNet Network

BOLD5K (Tags): tree, person, city.

Mini-Kinetics (Tags): music, tree, play.

WikiArt (Tags): tree, river, person.

Prosody (Tags): (no overlap).

BOLD5K (Captions): person, place, tree, hold, make, play, set, take, line, go.

Mini-Kinetics (Captions): person, play, someone, music, make, hold, go, move, take, individual, work, set, player.

WikiArt (Captions): make, line, work, person, go, tree, place.

Prosody (Captions): person, state, someone, make, go.

Shared Community Themes Among Multimodal and Free Association Networks

BOLD5K (Tags): [water, ocean, fish, sea, boat, beach, wet, ship], [car, travel, road], [animal, bird, dog, furry], [music, instrument, singer, song], [food, fruit, bread], [green, tree], [man, person, woman].

Mini-Kinetics (Tags): [water, ocean, beach], [girl, child, baby], [game, ball, sport, football, baseball, sports], [music, instrument, band, dance], [food, meat, chicken], [man, woman], [car, drive, ride], [green, tree].

WikiArt (Tags): [circle, square], [water, ocean, boat, beach], [love, girl, woman], [green, red], [food, bread], [anger]

Prosody (Tags): [anger], [happy], [sad, woman], [loud]

BOLD5K (Captions): [girl, man, person, woman, young], [see, look], [shirt, dress], [green, tree], [car, go, road], [house, home], [play, game, player]

Mini-Kinetics (Captions): [high, jump, hill], [water, ocean, air], [go, move, ride], [small, little], [music, play, instrument, dance, song], [game, ball, sport, player, hit], [shirt, head, leg].

WikiArt (Captions): [square, shape], [girl, sad, man, person, woman], [happy, nice], [green, tree].

Prosody (Captions): [anger], [girl, woman], [happy], [sad], [talk, word], [think, smart], [loud, sound].

Appendix K

Reaction Time, Node Degree, and Word Frequency

Table K1

Pearson correlation between network log-degree and reaction time in a lexical decision task.

Dataset	N_{shared}	r	p
BOLD5K (Tags)	3741	-0.335	$< 10^{-4}$
Mini-Kinetics (Tags)	1383	-0.194	$< 10^{-4}$
WikiArt (Tags)	1750	-0.215	$< 10^{-4}$
Prosody (Tags)	624	-0.174	$< 10^{-4}$
BOLD5K (Captions)	5767	-0.400	$< 10^{-4}$
Mini-Kinetics (Captions)	3325	-0.352	$< 10^{-4}$
WikiArt (Captions)	5492	-0.340	$< 10^{-4}$
Prosody (Captions)	2385	-0.254	$< 10^{-4}$
Associations	4913	-0.462	$< 10^{-4}$
WordNet	27990	-0.356	$< 10^{-4}$

Note: The measures are: the number of shared words between the network and the lexical decision task N_{shared}, Pearson correlation r between log-degree and reaction time, and the corresponding p-value p.

Table K2

Spearman correlation between network log-degree and reaction time in a lexical decision task after partialing out log-frequency.

Dataset	N_{shared}	ρ	p
BOLD5K (Tags)	3741	-0.135	$< 10^{-4}$
Mini-Kinetics (Tags)	1383	0.037	.174
WikiArt (Tags)	1750	-0.037	.118
Prosody (Tags)	624	-0.014	.736
BOLD5K (Captions)	5767	-0.151	$< 10^{-4}$
Mini-Kinetics (Captions)	3325	-0.084	$< 10^{-4}$
WikiArt (Captions)	5492	-0.050	$< 10^{-3}$
Prosody (Captions)	2385	-0.040	0.050
Associations	4913	-0.246	$< 10^{-4}$
WordNet	27990	-0.093	$< 10^{-4}$

Note: The measures are: the number of shared words between the network and the lexical decision task N_{shared}, Spearman correlation ρ between log-degree and reaction time after partialing out log-frequency (see Methods), and the corresponding p-value p (Pearson correlations are provided in Table K3).

Table K3

Pearson correlation between network log-degree and reaction time in a lexical decision task after partialing out log-frequency.

Dataset	N_{shared}	r	p
BOLD5K (Tags)	3741	-0.152	$< 10^{-4}$
Mini-Kinetics (Tags)	1383	-0.005	0.853
WikiArt (Tags)	1750	-0.065	0.006
Prosody (Tags)	624	-0.039	0.326
BOLD5K (Captions)	5767	-0.161	$< 10^{-4}$
Mini-Kinetics (Captions)	3325	-0.098	$< 10^{-4}$
WikiArt (Captions)	5492	-0.072	$< 10^{-4}$
Prosody (Captions)	2385	-0.056	0.006
Associations	4913	-0.244	$< 10^{-4}$
WordNet	27990	-0.109	$< 10^{-4}$

Note: The measures are: the number of shared words between the network and the lexical decision task N_{shared}, Pearson correlation r between log-degree and reaction time after partialing out log-frequency (see Methods), and the corresponding p-value p.

Appendix L

Demographic Information

Table L1

Demographic information for the different behavioral experiments.

Experiment	N	M_{age}	R_{age}	N_{male}	N_{female}	N_{other}	N_{na}
BOLD5K (Tags)	1223	40.0	19 - 77	554	410	1	258
Mini-Kinetics (Tags)	221	40.4	20 - 77	114	68	0	39
WikiArt (Tags)*	257	-	-	-	-	-	-
Prosody (Tags)	217	41.4	20 - 68	114	75	0	28
BOLD5K (Captions)	410	41.3	19 - 79	230	178	2	0
Mini-Kinetics (Captions)	196	41.0	23 - 78	110	86	0	0
WikiArt (Captions)	229	41.0	19 - 71	130	98	1	0
Prosody (Captions)	151	42.4	23 - 77	73	78	0	0
BOLD5K (Similarity)	345	40.9	19 - 85	186	157	2	0
Mini-Kinetics (Similarity)	284	40.8	23 - 78	166	118	0	0
WikiArt (Similarity)	343	41.0	19 - 77	191	151	1	0
Prosody (Similarity)	252	41.2	20 - 78	133	119	0	0

Note: The measures are: the number of participants N, the average age M_{age}, the age range R_{age}, the number of participants that self-identified as male N_{male}, the number of participants that self-identified as female N_{female}, the number of participants that self-identified as other N_{other}, and the number of participants for whom sex information was not available due to early termination N_{na} (e.g., if the participant provided two or more tags that were flagged by other participants; see Methods). *Demographic information not available due to a technical error during data export.

Appendix M

Additional Descriptive Statistics

Table M1

Additional descriptive statistics for the STEP-Tag datasets.

Dataset	# Stimuli	# Tags	Per Stim. (M)	Per Stim. (SD)	Rating (M)	Rating (SD)
BOLD5K	4916	6751	9.27	3.62	4.17	0.69
Mini-Kinetics	1000	2181	8.41	2.90	4.12	0.69
WikiArt	1000	2124	9.36	3.91	3.55	0.77
Prosody	1000	787	9.02	3.47	3.76	0.87

Note: The columns are: number of unique stimuli, number of unique tags generated, average (M) and standard deviation (SD) of tags per stimulus, and average rating score and standard deviation per tag.

Table M2

Additional descriptive statistics for the Free Caption datasets.

Dataset	# Stimuli	# Tags	Per Stim. (M)	Per Stim. (SD)
BOLD5K	4916	7767	15.96	4.56
Mini-Kinetics	1000	4261	28.10	6.88
WikiArt	1000	6730	50.67	8.07
Prosody	1000	3028	28.69	5.04

Note: The columns are: number of unique stimuli, number of unique tags generated, and average (M) and standard deviation (SD) of tags per stimulus.

Table M3

Additional descriptive statistics for the similarity datasets.

Dataset	# Stimuli	# Pairs	# Judgments	Per Pair (M)	Per Pair (SD)
BOLD5K	100	4950	29305	5.92	0.84
Mini-Kinetics	100	4950	24105	4.87	0.83
WikiArt	100	4950	29137	5.89	0.83
Prosody	100	4950	21384	4.32	0.81

Note: The columns are: number of unique stimuli, number of unique pairs, overall number of human similarity judgments, and average (M) and standard deviation (SD) of the number of judgments per pair.

Table M4

Distribution statistics for average pairwise similarity scores.

Dataset	Mean	SD	Min	Max	95% CI
BOLD5K	1.26	1.07	0.0	6.0	[0.00, 4.14]
Mini-Kinetics	1.28	1.31	0.0	6.0	[0.00, 5.20]
WikiArt	2.22	1.09	0.0	5.67	[0.40, 4.50]
Prosody	2.48	1.41	0.0	6.0	[0.20, 5.28]

Note: The statistics are: mean, standard deviation, min, max, and 95% confidence intervals.

References

Alstott, J., Bullmore, E., & Plenz, D. (2014). Powerlaw: A python package for analysis of heavy-tailed distributions. *PloS one*, *9*(1), e85777.

Baevski, A., Hsu, W.-N., Xu, Q., Babu, A., Gu, J., & Auli, M. (2022). Data2vec: A general framework for self-supervised learning in speech, vision and language. *International conference on machine learning*, 1298–1312.

Baevski, A., Zhou, Y., Mohamed, A., & Auli, M. (2020). Wav2vec 2.0: A framework for self-supervised learning of speech representations. *Advances in neural information processing systems*, *33*, 12449–12460.

Chen, S., Wang, C., Chen, Z., Wu, Y., Liu, S., Chen, Z., Li, J., Kanda, N., Yoshioka, T., Xiao, X., et al. (2022). Wavlm: Large-scale self-supervised pre-training for full stack speech processing. *IEEE Journal of Selected Topics in Signal Processing*, *16*(6), 1505–1518.

Clauset, A., Newman, M. E., & Moore, C. (2004). Finding community structure in very large networks. *Physical Review E—Statistical, Nonlinear, and Soft Matter Physics*, *70*(6), 066111.

Clauset, A., Shalizi, C. R., & Newman, M. E. (2009). Power-law distributions in empirical data. *SIAM review*, *51*(4), 661–703.

Devlin, J., Chang, M.-W., Lee, K., & Toutanova, K. (2018). Bert: Pre-training of deep bidirectional transformers for language understanding. *arXiv preprint arXiv:1810.04805*.

Dosovitskiy, A., Beyer, L., Kolesnikov, A., Weissenborn, D., Zhai, X., Unterthiner, T., Dehghani, M., Minderer, M., Heigold, G., Gelly, S., et al. (2020). An image is worth 16x16 words: Transformers for image recognition at scale. *arXiv preprint arXiv:2010.11929*.

Fan, H., Murrell, T., Wang, H., Alwala, K. V., Li, Y., Li, Y., Xiong, B., Ravi, N., Li, M., Yang, H., et al. (2021). Pytorchvideo: A deep learning library for video

understanding. *Proceedings of the 29th ACM international conference on multimedia*, 3783–3786.

Feichtenhofer, C. (2020). X3d: Expanding architectures for efficient video recognition. *Proceedings of the IEEE/CVF conference on computer vision and pattern recognition*, 203–213.

Feichtenhofer, C., Fan, H., Malik, J., & He, K. (2019). Slowfast networks for video recognition. *Proceedings of the IEEE/CVF international conference on computer vision*, 6202–6211.

Gao, T., Yao, X., & Chen, D. (2021). Simcse: Simple contrastive learning of sentence embeddings. *arXiv preprint arXiv:2104.08821*.

Hsu, W.-N., Bolte, B., Tsai, Y.-H. H., Lakhotia, K., Salakhutdinov, R., & Mohamed, A. (2021). Hubert: Self-supervised speech representation learning by masked prediction of hidden units. *IEEE/ACM transactions on audio, speech, and language processing*, *29*, 3451–3460.

Liu, Z., Mao, H., Wu, C.-Y., Feichtenhofer, C., Darrell, T., & Xie, S. (2022). A convnet for the 2020s. *Proceedings of the IEEE/CVF conference on computer vision and pattern recognition*, 11976–11986.

Marjieh, R., Van Rijn, P., Sucholutsky, I., Sumers, T., Lee, H., Griffiths, T. L., & Jacoby, N. (2023). Words are all you need? language as an approximation for human similarity judgments. *The Eleventh International Conference on Learning Representations*.

Speer, R., Chin, J., & Havasi, C. (2017). Conceptnet 5.5: An open multilingual graph of general knowledge. *Proceedings of the AAAI conference on artificial intelligence*, *31*(1).

Wightman, R. (2019). Pytorch image models. https://doi.org/10.5281/zenodo.4414861

Yang, Y.-Y., Hira, M., Ni, Z., Astafurov, A., Chen, C., Puhrsch, C., Pollack, D., Genzel, D., Greenberg, D., Yang, E. Z., et al. (2022). Torchaudio: Building blocks

for audio and speech processing. *ICASSP 2022-2022 IEEE International Conference on Acoustics, Speech and Signal Processing (ICASSP)*, 6982–6986.
